# QTL mapping and successful introgression of the spring wheat-derived QTL *Fhb1* for Fusarium head blight resistance in three European triticale populations

**DOI:** 10.1007/s00122-019-03476-0

**Published:** 2020-01-20

**Authors:** Marine Ollier, Vincent Talle, Anne-Laure Brisset, Zoé Le Bihan, Simon Duerr, Marc Lemmens, Ellen Goudemand, Olivier Robert, Jean-Louis Hilbert, Hermann Buerstmayr

**Affiliations:** 1 Department of Agrobiotechnology, IFA-Tulln, Institute of Biotechnology in Plant Production, BOKU-University of Natural Resources and Life Sciences Vienna, Konrad Lorenz Str. 20, 3430 Tulln, Austria; 2 EA 7394, USC INRA 1411, Institut Charles Viollette (ICV), Agro-Food and Biotechnology Research Institute, Université de Lille, INRA, ISA, Univ. Artois, Univ. Littoral Côte d’Opale, Cité Scientifique, 59655 Villeneuve d’Ascq, France; 3 Florimond-Desprez Veuve & Fils SAS, 3 rue Florimond-Desprez, BP 41, 59242 Cappelle-en-Pévèle, France; 4 Present Address: Saatzucht Donau GmbH & Co KG, Breeding Station, Reichersberg, Austria; 5 Present Address: Bayer Crop Science, Le petit Boissay, Toury, France

**Keywords:** Triticale, Fusarium head blight, Resistance breeding, WKS, QTL, Marker, GBS, SSR, *Fhb1*, *Ddw1*

## Abstract

**Key message:**

The spring wheat-derived QTL *Fhb1* was successfully introgressed into triticale and resulted in significantly improved FHB resistance in the three triticale mapping populations.

**Abstract:**

Fusarium head blight (FHB) is a major problem in cereal production particularly because of mycotoxin contaminations. Here we characterized the resistance to FHB in triticale breeding material harboring resistance factors from bread wheat. A highly FHB-resistant experimental line which derives from a triticale × wheat cross was crossed to several modern triticale cultivars. Three populations of recombinant inbred lines were generated and evaluated in field experiments for FHB resistance using spray inoculations during four seasons and were genotyped with genotyping-by-sequencing and SSR markers. FHB severity was assessed in the field by visual scorings and on the harvested grain samples using digital picture analysis for quantifying the whitened kernel surface (WKS). Four QTLs with major effects on FHB resistance were identified, mapping to chromosomes 2B, 3B, 5R, and 7A. Those QTLs were detectable with both *Fusarium* severity traits. Measuring of WKS allows easy and fast grain symptom quantification and appears as an effective scoring tool for FHB resistance. The QTL on 3B collocated with *Fhb1*, and the QTL on 5R with the dwarfing gene *Ddw1*. This is the first report demonstrating the successful introgression of *Fhb1* into triticale. It comprises a significant step forward for enhancing FHB resistance in this crop.

**Electronic supplementary material:**

The online version of this article (10.1007/s00122-019-03476-0) contains supplementary material, which is available to authorized users.

## Introduction

Fusarium head blight (FHB), caused mainly by *Fusarium graminearum* and *Fusarium culmorum* (Bai and Shaner 1994, 2004; Mesterházy et al. 2005; Ruckenbauer et al. 2001; Schroeder and Christensen 1963), is considered a disease of major importance in most areas of the world where wheat and other small-grain cereals are grown. FHB can infect all members of the *Gramineae* and may significantly damage cereal crop within a few weeks after flowering (McMullen et al. 1997; Parry et al. 1995; Windels 2000). In addition to yield losses, the contamination of the harvest by secondary fungal metabolites, known as mycotoxins, can devalue or even render the crop unsuitable for food and feed uses (D’Mello et al. 1999; Desjardins 2006; Kotowicz et al. 2014; Mesterházy et al. 1999; Windels 2000). Mycotoxin contaminations in cereals for downstream processing, such as milling, production of bioethanol or brewing, are even more crucial since toxins tend to concentrate in the by-products, such as bran and distiller’s dried grains with solubles (DDGS) that are commonly used as animal feed (Pinotti et al. 2016). Among the numerous Fusarium mycotoxins, deoxynivalenol (DON) and its derivatives are the most prevalent ones (Joffe 1986; Rotter 1996). They are harmful to both humans and livestock when ingested (Ghareeb et al. 2015, Gilbert and Tekauz 2000; Sobrova et al. 2010). Numerous countries have established guidelines or regulations for maximum DON content in cereals and cereal products in order to ensure the safety of food and feed (Guidance for Industry and FDA 2010; Van Egmond and Jonker 2004). As an example, the European authorities have set a limit of 1.25 mg/kg DON in unprocessed cereals other than durum wheat, oats and maize (Commission Regulation (EC) No. 1126/2007). Limiting Fusarium head blight development is the key for reducing mycotoxin contamination in cereal products. Chemical control measures are only partly effective in controlling *Fusarium* in small-grain cereals (Mankeviciene et al. 2008; Šíp et al. 2010; Stack 2000), and the use of FHB-resistant cultivars combined with appropriate crop management practices is considered the most efficient method for managing this disease (Buerstmayr et al. 2009; Parry et al. 1995). Therefore, breeding cereal cultivars which are resistant to FHB and to the associated mycotoxin contaminations plays a crucial role for an integrated and sustainable management of this disease.

Genetic resistance to FHB in small grains is non-race specific, quantitatively inherited, i.e., controlled by several genes with effects ranking from low to high and has a moderate-to-high heritability depending on population (Bai and Shaner 1994, Van Eeuwijk et al. 1995). Several types of mechanism underlying the genetic resistance have been described (Mesterházy 1995; Mesterházy et al. 1999; Miller et al. 1985; Schroeder and Christensen 1963). Resistance to initial infection (type 1) and resistance to fungal spread from an infected floret along the rachis (type 2) were first described by Schroeder and Christensen (1963). The overall FHB resistance is termed ‘FHB severity in field’ in this publication. It is assessed by evaluating the proportion of infected spikelets on a whole plot basis after spray inoculation and is considered to reflect the genotypic response during natural epidemics. The number of infected spikelets can be directly correlated with the number of damaged kernels. Some genotypes can, however, show invasion of seeds without visible sign of damage on hulls (Schroeder and Christensen 1963). Scoring for additional types of resistance is therefore of high interest. Resistance to deoxynivalenol (DON) accumulation, also known as type 3 resistance (Miller et al. 1985), is of particular interest for breeding. Several methods exist to directly quantify the DON content of a grain sample (Koch 2004; Krska et al. 2007; Maragos and McCormick 2000; Saccon et al. 2017; Sinha et al. 1995). Determination of toxin content is, however, expensive and therefore scarcely performed on large sample numbers in breeding programs. Breeders favor instead visual scorings to estimate the proportion of Fusarium damaged kernels (FDK) also known as type 4 resistance (Mesterházy 1995). Previous studies have shown that the correlation between DON content and the proportion of FDK in a grain sample is generally higher than the correlation between DON content and FHB severity observed on spikes in the field (Buerstmayr and Lemmens 2015; Paul et al. 2005, 2006). Infected grains can be visually differentiated from healthy ones, because they tend to be smaller, shriveled and white to pale pink colored (Abramson et al. 1987; Mesterházy et al. 2005; Ruckenbauer et al. 2001). Although FDK is a widely used method, its scoring by visual inspection is subjective, time-consuming, and labor intensive. Instead of performing visual evaluations of the damaged kernels, measurements using digital image analysis have shown great promise, such as quantifying the whitened kernel surface (WKS). WKS was recently suggested as a fast, easy and reliable measurement of FHB severity on grains through digital picture analysis. Correlations between WKS and FDK are high, and correlations between WKS and DON content are in the same range as between FDK and DON content (Ollier et al. 2018).

Aside from the above-described resistance mechanisms, plant height, ear morphology, or earliness can also significantly influence resistance to FHB (Buerstmayr et al. 2011, 2012; Draeger et al. 2007; Kalih et al. 2014; Klahr et al. 2007; Mesterházy 1995; Paillard et al. 2004; Schmolke et al. 2008, Steiner et al. 2019; Boeven et al. 2016; Miedaner et al. 2017). The widely deployed Norin 10 semi-dwarfing *Rht* alleles, namely *Rht*-*B1b* and *Rht*-*D1b*, have been found associated with increased FHB susceptibility in bread wheat (Hilton et al. 1999; Mao et al. 2010; Miedaner and Voss 2008) and in durum wheat (Buerstmayr et al. 2012; Prat et al. 2017). Similarly, the dwarfing allele of the *Ddw1* gene commonly deployed in triticale germplasm and located on the rye chromosome 5R (Korzun et al. 1996) has been found to be related with increased FHB susceptibility in triticale (Kalih et al. 2014).

Triticale (x*Triticosecale* Wittmack) is the intergeneric amphidiploid between the female parent wheat (*Triticum* ssp.) and the male parent rye (*Secale* ssp.) with the first commercial varieties being released in the 1970s. Modern commercial varieties of this man-made crop have a genomic constitution of AABBRR with 2*n* = 6*x* = 42 chromosomes (Oettler 2005). They combine the high yield potential and good grain quality of wheat with winter hardiness and adaptation to unfavorable soils of rye (FAO 2004). Most of the produced grain is used on-farm as a feed grain, although triticale has shown great potential in biofuels (ethanol), organic and industrial chemicals, paper, the building and plastic industries and the beverage (beer) industry (FAO 2004). In 2017, it was cultivated on about 3.5 million ha in Europe where Poland, Belarus, Germany, and France are the main producers with 73% of the total European triticale acreage (FAOSTAT 2019). Triticale has shown high levels of disease resistance in the past, although with the increasing acreage in recent years FHB has become an important issue for farmers especially for pig and poultry production due to the risk for livestock of being fed with contaminated triticale grain (Goral et al. 2002; Murugesan et al. 2015; Pierron et al. 2016). The resistance of modern triticale varieties against FHB ranges approximately between its original parents wheat and rye (Kiecana et al. 1987; Langevin et al. 2004; Miedaner et al. 2001), allowing genetic improvement via resistance breeding by recurrent selection (Miedaner et al. 2004; Oettler and Wahle 2001). Winter triticale appears on average less susceptible to head blight than bread wheat, but there are large differences in resistance between specific triticale genotypes and even highly FHB susceptible triticale cultivars have been observed. This shows that studies on resistance of winter triticale should be conducted to preserve triticale’s reputation as a ‘healthy crop’ (Goral et al. 2002). However, relatively few studies have been conducted to understand FHB resistance in triticale and to elucidate its genetic architecture (Dhariwal et al. 2018; Galiano-Carneiro et al. 2019; Kalih et al. 2014, 2015; Miedaner et al. 2016). On the other hand, the different kinds of genetic resistance to FHB are relatively well characterized for bread wheat (Buerstmayr et al. 2009). Since most of the identified QTLs are located on the A and B genomes (Buerstmayr et al. 2009; Liu et al. 2009; Löffler et al. 2009), bread wheat represents a promising reservoir of resistance for triticale. Interspecific hybridization between wheat and triticale is furthermore a reliable method for transferring genetic information from one species to another and has been used to improve the resistance or the agronomic features of both crops, triticale and wheat (Hills et al. 2007; Lukaszewski and Gustafson 1983; Oettler 2005; Saulescu et al. 2011). The introgression of FHB-resistance QTLs from bread wheat into the genetic background of triticale could therefore be a promising strategy to broaden the genetic diversity of resistance factors in elite triticale germplasm.

Among the QTLs for FHB resistance identified in bread wheat, those on chromosomes 3BS (*Fhb1*) and 5AS (*Qfhs.ifa*-*5A*) are the most prominent ones (Buerstmayr et al. 2009). Both derive from the well-known resistance donor Sumai-3 (Buerstmayr et al. 1999; Waldron et al. 1999). *Fhb1* is a well-characterized QTL which has been validated in numerous studies and confers a high level of FHB resistance to fungal spreading (type 2 resistance) (Agostinelli et al. 2012; Anderson et al. 2001; Balut et al. 2013; Bourdoncle and Ohm 2003; Buerstmayr et al. 2002, 2003; Chen et al. 2006; Cuthbert et al. 2006; Jiang et al. 2007; Lemmens et al. 2005; McCartney et al. 2007; Prat et al. 2017; Shen et al. 2003; Waldron et al. 1999). *Qfhs.ifa*-*5A*, on the other hand, has been shown mainly to increase resistance to initial infection (type 1) (Buerstmayr et al. 2003; Lin et al. 2006; Xue et al. 2011) and is tightly associated with high anther extrusion in bread wheat (Steiner et al. 2019).

The impact of these two major QTLs on FHB resistance in triticale has, however, not been investigated until now. For this purpose, three related mapping populations were generated by crossing an FHB-resistant triticale pre-breeding line possessing *Fhb1* and *Qfhs.ifa*-*5A* with two current triticale cultivars and one F1 hybrid. These mapping populations were evaluated in replicated field trials under *Fusarium* inoculation in order to map, quantify and validate stable QTL for FHB resistance in the genetic background of modern triticale.

The aims of this study were thus (1) to get further insight into the genetic architecture of FHB resistance in triticale, (2) to examine the effect of *Fhb1* and *Qfhs.ifa*-*5A* in triticale genetic backgrounds and show the value of introgressing wheat resistance factors in elite triticale germplasms, (3) to investigate the association of plant height and FHB resistance with specific focus on the dwarfing gene *Ddw1*, (4) and finally to evaluate, in a breeding context, the potential of the WKS, a new method of FHB symptom measurement on grains by digital picture analysis.

## Materials and methods

### Plant materials

Three related mapping populations were developed from crosses between the FHB-resistant triticale line G8.06 and three triticale cultivars Tulus (T), Elpaso (E), and the F1 of Agostino × Grenado (AG), respectively. The crosses were carried out by Dr. Herbert Bistrich from the breeding company Saatzucht Donau GesmbH (Austria). F_2_ populations were returned to IFA-Tulln, and advanced to the F_4_ generation by single seed descent without intended selection. Seeds descending from single F_4_ spikes were bulk propagated and used as F_4:5_ lines for field tests in 2014 and 2015. F_4:5_ lines were propagated in microplots in 2015, and F_4:6_ lines used for the field trials in 2016 and 2017. Tulus is a variety bred by Nordsaat Saatzucht GmbH (Germany) and registered in 2008. Agostino is a variety bred by Lantmaennen SW Seed B.V. (Netherland) and registered in 2009. Elpaso and Grenado are both varieties bred by DANKO Hodowla Roslin sp. z o.o. (Poland) and registered in 2010 and 2004, respectively. Santop is a variety bred by Saatzucht Dr Hege GbRmbH (Germany) and registered in 1998. The triticale pre-breeding line G8.06 was developed at IFA-Tulln (Austria) through two generations of marker-assisted backcrossing of the highly FHB-resistant spring wheat line CM-82036 (Sumai-3 × Thornbird-S), which possesses the FHB-resistance QTLs *Fhb1* and *Qfhs.ifa*-*5A* (Buerstmayr et al. 2002, 2003) into the background of the triticale cultivar Santop (Hege Seeds, Germany). Line G8.06 was selected among ten BC2 lines as the one with the highest and most consistent level of FHB resistance in replicated field trials (data not shown). One hundred twenty F_4:5_ lines from each of the three populations were sown in the field and evaluated for *Fusarium* resistance and flowering time in 2014. Among those descendants, 92 lines were chosen from populations T and E and 91 from population AG for QTL mapping. Selection criteria were set to represent the whole range in FHB severity but avoiding visibly heterogeneous and very early and very late flowering lines. The objective was to keep the populations diverse for FHB symptom severity and to reduce the diversity in flowering time.

### Field trial and *Fusarium* infection

The three mapping populations and the parental lines were tested in repeated *Fusarium* inoculated field experiments at IFA-Tulln, Austria (48°19′05″N 16°04′08″E, 177 m above sea level) during four growing seasons from 2014 to 2017. Temperature and precipitation during each trial year are shown in Online resource 1. Experiments were laid out in randomized complete block design with two blocks per population. Plots consisted of double rows of 1 m length and 17 cm spacing. Sowing time was late October to early November in each season. The two blocks were sown 2 weeks apart. These staggered sowing dates led to slightly different flowering dates between the blocks. Management of the field trials was conducted following good agronomical practice as described in Buerstmayr et al. (2002). All experiments were spray inoculated with a motor-driven backpack sprayer in the late afternoons. The DON-producing *F. culmorum* isolate IFA104, at a conidial concentration of 5.0 × 10^4^ mL^−1^, was used in 2014 and 2015, and the isolate Fc91015, at a conidial concentration of 2.5 × 10^4^ mL^−1^, was used in 2016 and 2017. Inoculations were performed within each block on all plots, when 50% of the plants in the earliest plot of a block reached anthesis. Inoculations were repeated at 2-day intervals and ended 2 days after the last plot of the block flowered, resulting in up to six inoculum applications per block. At each inoculation cycle, about 100 mL m^−2^ of conidial suspension was sprayed onto the triticale heads. Inoculum suspension was prepared by using the protocol described in Buerstmayr et al. (2000). Aliquots of conidia stock solutions were stored at − 80 °C, then thawed at 37 °C and diluted with tap water to achieve the desired final spore concentration just prior to inoculation. An automatic mist-irrigation system, switched by leaf-wetness measurement, maintained humidity and kept the plants wet for 20 h after inoculation to facilitate spore germination and infection.

### FHB-resistance scoring

FHB severity was visually estimated as the percentage of infected spikelets within each plot on days 10, 14, 18, 22, and 26 after anthesis. The area under the disease progress curve (AUDPC) was calculated and used as an integrated measure of the overall disease severity as described by Buerstmayr et al. (2000). Plant height (PH) was measured in centimeters from the soil surface to the top of the head, excluding awns, and the date of flowering was recorded and converted into days after May 1 (Dmay) for all experimental plots.

*Fusarium* symptoms on grains were digitally assessed using the whitening kernel surface (WKS) trait evaluation as described in detail in Ollier et al. (2018) (Online resource 2). All plots were harvested at full ripening, using a plot combine harvester (Wintersteiger Nursery Master) set to low wind speed to avoid or reduce the loss of lightweight infected kernels. Twenty grams of grain from each seed sample was poured in bulk on a blue tinted paper and photographed under standardized light conditions. The red, green, and blue levels of each pixel (RGB levels) within a picture were analyzed using a script written in Python (Python Software Foundation, Inc. Python Language Reference, version 3.4.1, available at http://www.python.org). Pixels of each picture were separated into three categories based on their RGB levels: background, healthy grain, and diseased grain pixels. The WKS was evaluated as the percentage of diseased among all grain pixels. The differentiation between healthy and diseased kernel pixels was based on a blue-level limit determined through calibration as described in Ollier et al. (2018). This level was set to 150 for the three populations and all the analyses presented in this publication.

### Phenotypic data analysis

Statistical tests were performed for each population separately. A first analysis was performed for single experiments with a linear model of the form:

1 $$P_{ik} = \mu + G_{i} + R_{k} + e_{ik}$$

where *P*_*ik*_ is the phenotypic value, *µ* the population mean, *G*_*i*_ the effect of the *i*th genotype treated as fixed, *R*_*k*_ the random *k*th replicate effect, and e_ik_ the residual effect with $e \sim N\left( {0, \sigma_{e}^{2} } \right)$.

A combined analysis across experiments was then performed by fitting the linear model:

2 $$P_{ijk} = \mu + G_{i} + E_{j} + E_{j} \left( {R_{k} } \right) + GE_{ij} + e_{ijk}$$

where *P*_*ijk*_ is the phenotypic value, µ the population mean, *G*_*i*_ the effect of the *i*th genotype, *E*_*j*_ the effect of the *j*th experiment, *E*_*j*_(*R*_*k*_) the effect of the *k*th replicate nested within the *j*th experiment, *GE*_*ij*_ the *ij*th effect of the genotype-by-experiment interaction, and *e*_*ijk*_ designates the residual.

Best linear unbiased estimates (BLUEs) for the AUPDC, WKS, plant height, and flowering date of each line were derived from both models with experiment and replicate effects modeled as random, as was the genotype-by-experiment interaction, whereas the genotype effect was treated as fixed. Significance of genotypic effects was attested with both models and with all factors treated as fixed. For all statistical tests, the parental lines were excluded from the calculations. Finally, broad-sense heritability coefficients for each trait were derived from both models with all effects set as random and were calculated according to Holland et al. (2003):

3 $$H^{2} = \sigma^{2}_{\text{G}} /\left( {\sigma^{2}_{\text{G}} + \left( {\sigma^{2}_{{{\text{G}} \times {\text{E}}}} /e} \right) + \, \left( {\sigma^{2}_{\text{e}} /{\text{re}}} \right)} \right)$$

where *σ*_G_^2^ denotes the genotypic variance, *σ*_GxE_^2^ the genotype-by-experiment interaction variance, *σ*_e_^2^ the error variance that were determined by the restricted maximum likelihood (REML) method, *e* indicates the number of experiments, and re the total number of observation plots per line.

Statistical analysis was performed in R 3.3.2 (R Core Team 2016). All linear mixed and random models were fitted with the *lme4* package (Bates et al. 2015), while multiple comparisons of line means were performed with the Tukey’s range test as implemented in *agricolae* (Mendiburu 2015).

### Genotypic data

Genomic DNA was extracted from fresh leaves of 10 pooled plants of each F_4_ and parental lines using a CTAB-based procedure modified from Saghai-Maroof et al. (1984). High-density genotyping of all individuals was performed using genotyping-by-sequencing (GBS) with the DArTseq platform (DArT PL, Canberra, Australia). The markers identified by the DArTseq assay included SNP as well as presence–absence variations (PAV) (Li et al. 2015). The markers were filtered based on a call rate ≥ 95% and less than 20% missing data. For linkage map construction and QTL mapping of the AG population, which is a 3-way cross, only markers which were monomorphic among Grenado and Agostino (= homozygous) and polymorphic between Agostino/Greando and G8.06 were chosen. Markers showing significant segregation distortions (*p* < 0.10) were also discarded in all three populations. Finally, a total of 2216, 710, and 420 SNP were available for the T, AG, and E populations after quality filtering, while the number of PAV was slightly higher with 15124, 4092, and 6726 markers per population, respectively. In addition, all F_4_ and parental lines were genotyped with four simple sequence repeat (SSR) markers, *gwm493* and *gwm533* (Roeder et al. 1998) linked to *Fhb1* (McCartney et al. 2004), and *barc180* and *barc56* (Song et al. 2005*)* linked to *Qfhs.ifa*-*5A* (Buerstmayr et al. 2002). The analysis of SSR marker was done as described by Steiner et al. (2004). Agostino, Grenado, G8.06, and the 91 F_4_ of the AG population were finally genotyped with conserved ortholog set (COS) markers linked to the dwarfing gene *Ddw1* (Hackauf and Goldfisch pers. communication).

*Linkage maps construction* Cross-specific linkage maps of the AG and E populations were constructed with all available markers, codominant SNPs and dominant PAVs. The software CarthaGene 1.2.3 (De Givry et al. 2005) was selected to build the map due to its capacity to deal with dominant markers and with the residual heterozygosity in the F_4_ lines. Robust linkage groups were constructed using a maximum two-point distance of 50.0 cM (Haldane) and a minimum two-point LOD of 15.0. The markers in common with the triticale map provided by Tyrka et al. (2015) and with the wheat consensus map version 4 provided by DArT PL (Diversity Arrays Technologies, personal communication, 2016) were used as reference points for assigning linkage groups to specific chromosomes. Markers were then ordered through the initial framework mapping command *buildfw*. This incremental insertion procedure was set with a keep and an adding threshold of 3.0 LODs, starting the build process from an empty map. Finally, the genetic distances between markers in centimorgan (cM) were calculated using the Kosambi mapping function.

The T population displays 3 times more SNP markers than the two other populations. It was therefore possible to construct the cross-specific linkage map of this population based on SNP markers only and using the MSTmap algorithm (Wu et al. 2008) included in the R package ASMap V0.4 (Taylor and Butler 2015). The objective function was set to minimize the sum of recombination events between markers for map construction. Robust linkage groups where constructed using a *p* value threshold set to 1 × 10^−9^ in a first step, and the assignment of the linkage groups to chromosome was done as described above by comparing the location of markers to markers from the triticale map provided by Tyrka et al. (2015) and the wheat consensus map version 4 provided by DArT PL (Diversity Arrays Technologies, personal communication, 2016). Genotypic data were subsequently pooled on a chromosome basis and regrouped at a less stringent threshold using a p value of 1 × 10^−6^. Genetic distances were calculated with the Kosambi mapping function.

Consensus maps for the chromosomes 2B, 3B, 5A, 5R, and 7A, which appeared as of special interest in our study, were constructed across the three populations. All markers previously selected to construct the three cross-specific linkage maps and all additional high-quality SNP and PAV markers that were polymorphic in at least two populations were used. New marker ordering processes were run with CarthaGene 1.2.3 (De Givry et al. 2005) for each population and each of these five specific chromosomes. The generated cross-specific linkage maps of the three populations were chromosome-wise merged, while ensuring that the ordering of the markers in the individual linkage maps is preserved by using the R package LPmerge (Endelman and Plomion 2014). Genetic maps were finally drawn with MapChart (Voorrips 2002).

*QTL mapping* The calculated BLUEs from the analysis within individual experiments and across experiments were used for quantitative trait loci analyses that were performed for each trait separately. QTL mapping was first performed for each population individually with the previously described cross-specific maps by performing interval mapping and composite interval mapping via the multiple imputation method (Sen and Churchill 2001) as implemented in the R package *R/qtl* (Broman et al. 2003). The number of marker covariates was selected by a forward approach in the composite interval mapping, while setting a window size of 10 cM. LOD significance threshold for a type I error rate of α ≤ 0.05 was obtained for each trait and experiment based on a 1000 times replicated permutations test (Churchill and Doerge 1994), and significant QTLs were subsequently fitted in a multiple-QTL model. The existence of further QTL, the presence of QTL-by-QTL, or QTL-by-genetic background interaction was tested by using the *addqtl*, *addint*, and *addpair* functions, respectively (Broman et al. 2003). An ANOVA was conducted with the final multiple-QTL model to estimate the proportion of the phenotypic variance explained by all terms in the model. The percentage of phenotypic variance explained by each QTL as well as their LOD scores were estimated by a type III sum of squares test by dropping one QTL at a time and comparing the full model to the model with the omitted term. Confidence intervals were finally defined for each QTL by calculating a 1.5-LOD support interval.

Thereafter, multi-parent population QTL mapping was realized to increase the power of QTL detection and compare the effects of QTLs detected in cross-specific models (Blanc et al. 2006; Li et al. 2005). The combined analysis of the three related mapping populations was performed by using the methodology outlined by Garin et al. (2017) with a focus on the parental and bi-allelic models. A parental model assumes the contribution of one unique allele per parental line. In related populations, the contribution of each cross-specific parent may differ characterizing the relative instability of the QTL in different genetic backgrounds. A bi-allelic model is based on the identical by state (IBS) assumption of each SNP, assuming that the same marker score corresponds to the same allelic state. The bi-allelic model is therefore similar to models used for genome-wide association mapping and allows a global characterization of the QTL alleles based on all available information. The detection of QTL in related populations, with both, parental and bi-allelic models, is only possible for QTL with a relatively small QTL x background interaction. QTLs were detected by performing simple interval mapping (SIM) and composite interval mapping (CIM) with both the parental and bi-allelic models with a homogeneous residual variance using the previously generated consensus map. For composite interval mapping, a maximum of one cofactor was selected per chromosome when being above the significance threshold of –log_10_(p value) = 3. The threshold for declaring significance of a marker–trait association has been empirically determined by using the 95% quantile value from a null distribution representing the maximum genome-wide significance values obtained from 1000 permutations. The effects of the QTL alleles and the percentage of the phenotypic variance explained by each QTL were estimated using a linear model including all significant QTL positions, whereas confidence intervals were defined for each QTL by calculating a 1.5-log_10_(p value) drop-off interval.

Robustness of QTL was evaluated employing a fivefold cross-validation (CV), replicated 20 times, following a modified algorithm of Utz et al. (2000) adapted to the multi-parent populations context (Garin et al. 2017). Briefly, five subsets were generated within each cross with one subset used as validation set and the remaining subsets as training set at a time. The training set was used to detect QTL and the proportion of phenotypic variance explained by the detected QTL in the training set *pTS* was saved. The detected QTLs and their estimated effects were then used to predict the phenotypic values of the validation set with *pVS* representing the square correlation between the predicted and observed phenotypic values. The bias was calculated by *1*-*(pVS/pTS)* in order to get some insight into the stability of the estimated QTL effects. All multi-parent population QTL mapping analyses were performed with the R package *mppR* (Garin et al. 2017).

## Results

### Trait variations and correlations

**Fig. 1:**
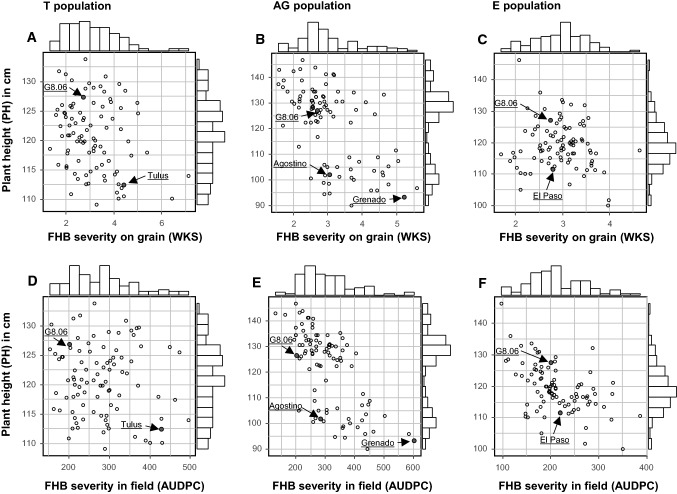
Scatter plots and marginal histograms of frequency distribution of BLUEs for: FHB severity on grains (WKS) against plant height (cm) for **a** the T population; **b** the AG population; **c** and the E population; and for FHB severity in field (AUDPC) again plant height (cm) for **d** the T population; **e** the AG population; **f** and the E population. Parents are indicated by arrows

Table 1 summarizes mean values of the parents, means and ranges of the populations, least significant differences, and broad-sense heritabilities for FHB severity in field (AUDPC) and on grains (WKS), plant height (PH), and flowering date, with variance component estimates available in Online resource 3. For all traits, significant genotypic effects were revealed, and continuous distributions were displayed within the three triticale populations, except for plant height in the AG population, which showed a bimodal frequency distribution (Fig. 1). The average FHB severity of the three populations was significantly lower in the E population than in the T, and AG populations, and the disease pressure was significantly different among years, with the 2016 experiment showing higher symptoms, followed by the 2014, 2017, and 2015 experiments. Transgressive segregation toward resistance was observed in all populations and was statistically significant for the T and AG populations, but not for the E one. Significant differences in plant height were observable among the parents of each population, whereas no such differences were detected for flowering date. For both traits, no transgressive segregation was observed. Correlations between AUPDC and WKS ranged between *r* = 0.61 and *r* = 0.78 for the three populations. Plant height (PH) was positively correlated with both FHB-resistance traits within the AG population, where taller plants showed significantly lower FHB severity. Correlations between plant height and FHB-resistance traits were lower in the T and E populations and did not exceed *r* = 0.5. Correlation coefficients between FHB-resistance traits and flowering date remained very low and varied between *r* = − 0.20 and *r* = 0.39 without revealing a clear pattern (Table 2).

**Table 1:** Means of parents and mean, minimum, and maximum values of populations, least significant differences at *α* < 0.05 (LSD_0.05_), and broad-sense heritability coefficient (H^2^) or repeatability of analyzed traits

	Parents					Population				
	G8.06	Tulus	Agostino	Grenado	El Paso	*T*				
						Mean	Min	Max	LSD_0.05_	H^2^
*FHB severity in field (AUDPC)*										
Overall mean	202	429	267	602	220	280	153	495	113	0.73
2014	115	322	126	696	119	148	34	377	72	0.83^b^
2015	42	106	63	189	91	58	4	296	50	0.81^b^
2016	607	941	752	1247	570	781	341	1416	331	0.51^b^
2017	42	346	129	277	101	133	17	623	117	0.82^b^
*FHB severity on grain (WKS)*										
Overall mean	2.71	4.43	3.06	5.22	2.79	3.10	1.32	7.13	1.09	0.87
2014	2.49	6.16	3.36	4.59	1.81	3.33	0.86	8.93	1.43	0.97^b^
2015	2.64	3.9	2.30	5.26	2.93	2.81	1.02	9.25	1.04	0.95^b^
2017	2.99	3.3	3.51	5.80	3.62	3.15	1.07	8.53	1.12	0.96^b^
Flowering date^a^	29.8	29.5	29.3	30.5	29.5	29.9	27.4	31.6	1.1	0.86
Plant height (cm)	127	112	102	93	112	121	109	134	6	0.88

	AG					E				
	Mean	Min	Max	LSD_0.05_	H^2^	Mean	Min	Max	LSD_0.05_	H^2^
*FHB severity in field (AUDPC*										
Overall mean	303	130	584	116	0.77	216	98	450	90	0.74
2014	186	43	498	90	0.88^b^	157	52	745	118	0.50^b^
2015	74	8	235	78	0.43^b^	82	4	517	81	0.68^b^
2016	840	252	1612	302	0.67^b^	537	153	1193	243	0.60^b^
2017	113	6	407	71	0.88^b^	86	10	333	65	0.80^b^
*FHB severity on grain (WKS)*										
Overall mean	3.04	1.57	5.59	0.85	0.89	2.98	1.69	6.69	0.71	0.86
2014	2.95	1.10	5.64	1.14	0.92^b^	2.88	1.29	8.12	0.92	0.96^b^
2015	2.88	0.81	7.63	1.26	0.94^b^	2.64	1.28	6.32	0.82	0.94^b^
2017	3.28	1.17	8.10	0.91	0.98^b^	3.41	1.53	7.34	0.98	0.94^b^
Flowering date^a^	29.4	27.9	31.8	1.1	0.56	29.6	27.5	31.5	1	0.83
Plant height (cm)	122	90	147	7	0.97	119	100	146	5	0.93

^a^Number of days from May 1 to anthesis

^b^Repeatability, means based on two replications

**Table 2:** Pearson correlation coefficients between FHB severity in field (AUDPC), FHB severity on grain (WKS), plant height (PH), and flowering date (days after May 1) for the overall means

	WKS			Plant height			Flowering date		
Population	*T*	AG	*E*	*T*	AG	*E*	*T*	AGp	*E*
AUDPC	0.78***	0.75***	0.61***	− 0.10 n.s	− 0.67***	− 0.48***	0.08 n.s	0.23*	− 0.14 n.s
WKS				− 0.25*	− 0.59***	− 0.14 n.s	0.04 n.s	0.39***	− 0.20 n.s
Plant height							0.25**	− 0.32**	0.10 n.s

*p < 0.05

**p < 0.01

***p < 0.001

*n.s.* nonsignificant

### Linkage maps

The number of markers within maps for the T, AG, and E populations was reduced to 1036, 432, and 430 unique loci with total map lengths of 2908, 2666, and 4324 cM per population. The average marker distance amounted 2.7, 6.5, and 10.5 cM for the T, AG, and E populations, respectively. Linkage groups were obtained for all chromosomes, except 7R and 2R for the T and AG populations, and 7R and 3R for the E population. Consensus maps built on the three populations for the chromosomes 2B, 3B, 5A, 5R, and 7A contained between 68 and 104 markers with an average space between two markers between 1.6 and 4.7 cM. For reading ease, only selected markers are displayed together with the QTL mapping results (Online Resource 4), while more detailed information concerning all mapped markers and their positions can be found in Online Resource 5.

### QTL analysis for flowering date and plant height

Multiple QTLs for flowering date were detected with cross-specific models on 4A and 5R for the T population, 3A and 5R for the AG population, and 4A, 6B, and 7A for the E population (Table 3, Online Resource 4). Colocalization of QTL for anthesis date and plant height was found only on chromosome 5R in the AG population. The QTL mapped to marker positions *Xiac129* and *Xiac130* flanking the dwarfing gene *Ddw1.* In the AG population, this QTL accounted for 78% and 25% of the variation for plant height and flowering date, respectively (Table 3), corresponding to an average height decrease of 28 cm and an average delay of flowering of 1 day. The use of a parental model, when performing an analysis on the three populations together with the consensus map, confirmed the effect of *Ddw1* in the AG population (Table 4). Additional QTLs for plant height were detected with cross-specific models on 5A and 6A for the T population, 2B and 5A for the AG population, and 5A and 5B for the E population. The common parent G8.06 contributed the tall allele for all of them except for the QTL on 2B detected in the AG population (Table 3, Online Resource 4). Significant epistatic interactions were observed for the plant height QTL on 5R and 2B in the AG population, and for the 5A and 5B QTL in the E population, explaining 1.4% and 11.5% of the phenotypic variance in their respective populations (Table 3). Three different QTLs, corresponding to three different positions, were characterized on chromosome 5A by the previously described parental model. The plant height QTL previously found on the chromosome 2B in the AG population was, however, not detected by the parental model.

**Table 3:** Locations and estimates of QTL for plant height (cm) and flowering date (days after May 1) on the cross-specific maps using cross-specific models run with the *R/QTL* package

Population	chr	Pos (cM)	Closest marker	Add^a^	%PV^b^	LOD^c^	Range^d^
*Plant height*							
AG	2B	113	*8535079*	-2.1	5.0	11.2	150.0
T	5A	100	*4211970|F|0*-*17:G *> *C*-*17:G *> *C*	2.5	16.5	5.0	28.0
AG	5A	15	*3615965*	4.5	3.8	9.1	12.0
E	5A	292	*4559414*	2.8	29.8	8.3	12.0
E	5B	188	*4369389|F|0*-*6:G *> *A*-*6:G *> *A*	0.4	27.4	7.8	31.3
E	5A x 5B	–	–	–	11.5	3.7	–
AG	5R	19	*Xiac129*	14.2	77.7	50.6	8.0
AG	5R x 2B	–	–	–	1.5	4.1	–
T	6A	103	*4339927|F|0*-*26:G *> *T*-*26:G *> *T*	3.3	28.7	8.0	11.3
*Flowering date*							
AG	3A	17	*10503667*	-0.32	12.4	4.5	91.0
T	4A	12	*8531145|F|0*-*12:G *> *A*-*12:G *> *A*	-0.48	9.9	4.1	24.0
E	4A	146	*10514293*	-0.46	11.4	3.9	34.0
T	5R	568	*3613461|F|0*-*15:T *> *C*-*15:T *> *C*	-0.52	7.2	3.0	12.0
AG	5R	19	*Xiac129*	-0.49	24.5	8.1	9.0
E	6B	196	*8512302|F|0*-*65:T *> *C*-*65:T *> *C*	-0.41	13.7	4.6	55.0
E	7A	281	*4210643|F|0*-*29:A *> *G*-*29:A *> *G*	0.37	15	5.0	110.0

^a^Positive additive effects denote trait-increasing effect of the G8.06 allele; additive effects were estimated as half the difference between phenotype averages for the homozygotes

^b^Percentage of phenotypic variance explained by the QTL

^c^LOD (logarithm of the odds) above LOD threshold at the 0.05 level of probability obtained through a 1000-iteration permutation test

^d^Range of the confidence interval position for the QTL

**Table 4:** Locations and estimates of QTL for plant height (cm) on the consensus map, including chromosomes 2B, 3B, 5A, 5R, 7A, and using a parental model run on all the lines from the three mapping populations with the *mppR* package

Chr	Closest marker	%PV^a^	LOD^b^	Pos^c^	Range^d^	Parent	Effect	*T* test^e^
5R	*Xiac129*	53.3	36.0	79.4	7.9	Tulus	− 0.5	n.s
						F1(Agos´xGren´)	− 14.1	***
						ElPaso	0.1	n.s
5A	*3622789|F|0*-*8:G *> *A*-*8:G *> *A*	1.9	3.3	71.9	66.5	Tulus	0.2	n.s
						F1(Agos´xGren´)	− 1.7	n.s
						ElPaso	5.4	***
5A	*4211970|F|0*-*17:G *> *C*-*17:G *> *C*	1.4	4.4	106.3	29.6	Tulus	− 1.9	*
						F1(Agos´xGren´)	− 0.9	n.s
						ElPaso	− 2.1	**
5A	*3619312|F|0*-*12:G *> *C*-*12:G *> *C*	3.0	5.5	164.2	6.9	Tulus	− 1.6	.
						F1(Agos´xGren´)	− 3.6	***
						ElPaso	− 0.9	n.s

^a^Percentage of phenotypic variance explained by the QTL

^b^LOD (logarithm of the odds) above LOD threshold at the 0.05 level of probability obtained through a 1000-iteration permutation test

^c^Best estimated position for the QTL in cM on the consensus Map

^d^Range of the confidence interval position for the QTL

^e^Student’s *T* tests results indicating when the tested parental effect is significantly different from the effect of the shared parent

*p* < 0.10

* *p* < 0.05

** *p* < 0.01

*** *p* < 0.001

*n.s.* nonsignificant

### QTL analysis for FHB severity in field and on grains

QTLs for FHB severity (AUDPC and WKS) were detected with cross-specific models on 2B, 3B, 6A, 6B, and 7B for the T population, 3B, and 5R for the AG population, and 6B and 7A for the E population. For all these QTLs, except the ones on 6B, the alleles from resistance donor parent G8.06 were associated with an increased FHB resistance (Table 5). Among all detected QTLs, those on 2B and 3B for the T population, 3B and 5R for the AG population, and 7A for the E population explained the largest proportion of phenotypic variance in their respective populations and were detected in all years with both traits, AUDPC and WKS.

**Table 5:** Locations and estimates of QTL for FHB severity (AUDPC and WKS) on the cross-specific maps using cross-specific models run with the *R/QTL* package

Trait	Population	chr	Pos (cM)	Closest marker	Add^a^	%PV^b^	LOD^c^	Range^d^	Validity per year
AUDPC	T	2B	58	*10517361|F|0*-*33:T *> *C*-*33:T *> *C*	43.40	26.2	12.5	33.6	All years
WKS	T	2B	58	*10517361|F|0*-*33:T *> *C*-*33:T *> *C*	0.60	14.5	7.2	32.0	2014, 2017
AUDPC	T	3B	78	*14479678|F|0*-*40:G *> *C*-*40:G *> *C*	38.09	21.7	10.8	32.1	All years
WKS	T	3B	78	*14479678|F|0*-*40:G *> *C*-*40:G *> *C*	0.60	29.7	12.7	52.0	All years
AUDPC	AG	3B	39	*gwm533*	44.04	14.0	4.8	72.4	All years
WKS	AG	3B	39	*gwm533*	0.48	14.1	5.1	52.0	All years
AUDPC	AG	5R	19	*Xiac129*	50.42	27.6	8.5	14.0	All years
WKS	AG	5R	19	*Xiac129*	0.57	30.2	9.6	8.0	All years
AUDPC	T	6A	40	*3605407|F|0*-*32:G *> *A*-*32:G *> *A*	7.39	10.2	5.8	72.9	All years
AUDPC	T	2B x 6A	–	–	–	6.7	4.0	–	2016
WKS	T	6B	29.3	*3619611|F|0*-*12:A *> *G*-*12:A *> *G*	− 0.19	7.5	4	24.4	2015, 2017
AUDPC	E	6B	114	*4369576|F|0*-*15:G *> *T*-*15:G *> *T*	− 23.09	14.9	3.8	136.0	All years
AUDPC	E	7A	198	*8514068*	24.64	18.9	4.7	12.0	All years
WKS	E	7A	198	*8514068*	0.33	19.6	4.4	26.0	All years
AUDPC	T	7B	16	*3043611|F|0*-*39:T *> *C*-*39:T *> *C*	31.09	16.3	8.6	80.0	All years
AUDPC	T	3B x 7B	–	–	–	12.2	6.8	–	All years
WKS	T	7B	16	*3043611|F|0*-*39:T *> *C*-*39:T *> *C*	0.32	8.3	4.4	82.0	2017
WKS	T	3B x 7B	–	–	–	7.2	3.9	–	2017

^a^Positive additive effects denote trait-increasing effect of the G8.06 allele; additive effects were estimated as half the difference between phenotype averages for the homozygotes

^b^Percentage of phenotypic variance explained by the QTL

^c^LOD (logarithm of the odds) above LOD threshold at the 0.05 level of probability obtained through a 1000-iteration permutation test

^d^Range of the confidence interval position for the QTL

**Fig. 2:**
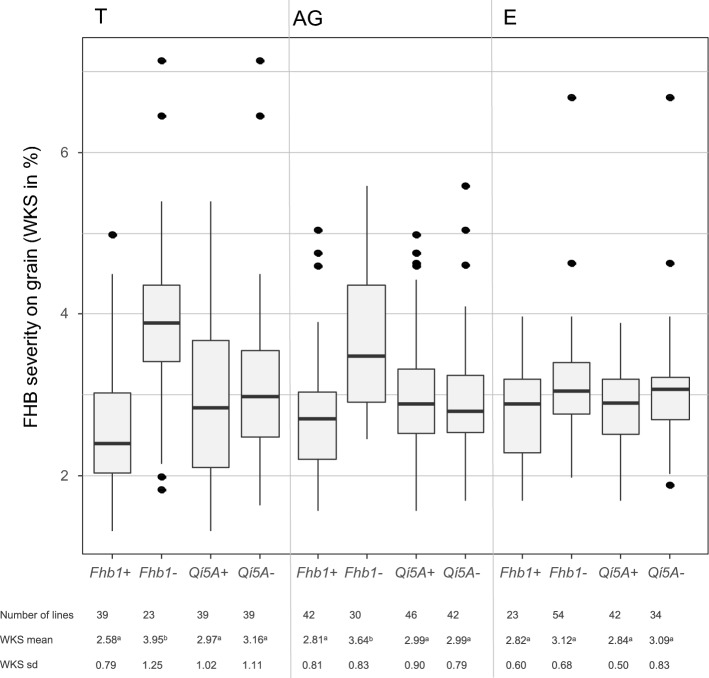
Box plot distributions of F_4_ according to their alleles at *Fhb1* and *Qfhs.ifa*-*5A* loci for the three tested populations based on BLUEs of FHB severity on grain (WKS). BLUEs were calculated across all experiments. Medians are indicated by solid lines, and points represent outliers. For each subgroup, the number of lines, mean values, and standard deviations FHB severity on grain (WKS) are indicated. Values followed by different letters are significantly different (*p* < 0.05) based on the Tukey test performed on each population independently

Markedly, the QTL detected on chromosome 3B mapped to marker positions *gwm493* and *gwm533*, which flank the position of the introgressed *Fhb1* locus from hexaploid wheat. *Fhb1* passed the significance threshold with cross-specific models across all experiments in the T and AG populations but not in the E one. The QTL was detected with both the parental and bi-allelic models (Table 6), and its stable effect was confirmed by cross-validation (Table 7), where it was significant in 96 out of 100 repetitions. Moreover, the higher detection power of the parental model allowed identifying a significant effect for *Fhb1* in all three populations, including the E population. The resistant allele of the QTL led to an average reduction of FHB symptom severity of 25%, 28%, 9% in field, and of 35%, 30%, and 8% on grains, for the T, AG, and E populations, respectively (Fig. 2, Online Resource 6). These substantial differences in the level of expression of the QTL among populations are characteristic for a QTL x genetic background interaction, which could partially be explained by the presence of epistatic interactions in this study. In the T population, a significant interaction was detected with the cross-specific model between *Fhb1* and the QTL on 7B. The genotypes carrying the G8.06 allele for both the 3B and 7B QTLs were significantly more resistant than genotypes presenting other allele combinations. In the T population cross-specific model, this interaction explained 12% of the global phenotypic variance in field and 7% on grains.

**Table 6:** Locations and estimates of QTL for AUDPC on the consensus map, including chromosomes 2B, 3B, 5A, 5R, 7A, and using bi-allelic and parental models run on all the lines from the three mapping populations with the *mppR* package

Chr	Model	Closest marker	%PV^a^	LOD^b^	Pos^c^	Range^d^	Parent	Effect	*T* test^e^
2B	Parental	*10517361|F|0*-*33:T *> *C*-*33:T *> *C*	9.4	6.6	144	14.1	Tulus	38.6	***
							F1(Agos´xGren´)	− 13.2	n.s
							ElPaso	− 29.5	**
	Bi-allelic	*11911490|F|0*-*41:G *> *T*-*41:G *> *T*	10.5	6.9	149.7	1.3	Tulus	39.9	***
							F1(Agos´xGren´)	0.0	n.s
							ElPaso	0.0	n.s
3B	Parental	*10524243|F|0*-*32:G *> *A*-*32:G *> *A*	14.7	8.9	59.7	15.5	Tulus	44.8	***
							F1(Agos´xGren´)	30.9	***
							ElPaso	22.4	**
	Bi-allelic	*14479870|F|0*-*26:A *> *T*-*26:A *> *T*	9.7	7.6	67.4	20.9	Tulus	28.6	***
							F1(Agos´xGren´)	28.6	***
							ElPaso	28.6	***
5R	Parental	*Xiac129*	8.1	6.3	79.4	11.3	Tulus	5.3	n.s
							F1(Agos´xGren´)	52.8	***
							ElPaso	14.0	.
	Bi-allelic	–	–	–	–	–	–	–	–

^a^Percentage of phenotypic variance explained by the QTL

^b^LOD (logarithm of the odds) above LOD threshold at the 0.05 level of probability obtained through a 1000-iteration permutation test

^c^Best estimated position for the QTL in cM on the consensus Map

^d^Range of the confidence interval position for the QTL

^e^Student’s *T* tests results indicating when the tested parental effect is significantly different from the effect of the shared parent

*p* < 0.10

* *p* < 0.05

** *p* < 0.01

*** *p* < 0.001

*n.s*. nonsignificant

**Table 7:** Confirmation per cross-validation of the QTL with the major effect on the resistance presented in Table 6

Chr	Model	Pos^a^	N^b^	p.Ts^c^	p.Vs^d^	Bias^e^
2B	Parental	144	63	9.4	6.5	0.3
	Bi-allelic	149.7	76	10.4	8.6	0.2
3B	Parental	59.7	96	14.4	10.7	0.3
	Bi-allelic	67.4	41	9.5	7.7	0.2
5R	Parental	83.3	47	9.0	5.1	0.4
	Bi-allelic	–	–	–	–	–

^a^Best estimated position for the QTL in cM on the consensus Map

^b^Number of occurrences of the QTL apparition across the 100 repetitions

^c^Percentage of phenotypic variance explained by the QTL in the global training set gathering the training sets of each cross

^d^Weighted average, accounting for the cross-sizes, of the within cross-values of the squared Pearson correlation between the observed and predicted phenotype values in the validation set

^e^Bias = 1 − (pVs/pTs), Measure of the relative difference between pTs and pVs. More the bias is close to 0, more the QTL is stable

The QTL *Qfhs.ifa*-*5A* from hexaploid wheat was also introgressed into the resistant triticale parent G8.06 and therefore segregating in all three mapping populations. However, none of the markers near this locus was found associated with FHB symptom severity with any of the tested models (Fig. 2, Online Resource 6).

The FHB-resistance QTL detected with cross-specific models on chromosome 5R in the AG population mapped to marker positions *Xiac129* and *Xiac130* which flank the dwarfing gene *Ddw1.* In this population, it exhibited a major effect on resistance with an average symptom severity reduction of 26% in field and of 31% on grains, with the tall allele enhancing resistance. The analysis performed on the three populations together with the consensus map and a parental model confirmed the effect of *Ddw1* in the AG population (Table 6), and the employed cross-validation tests showed an intermediate level of stability (Table 7) of this QTL which was significant in 47 out of 100 repetitions. No epistatic interaction was identified with this QTL, neither with the other QTLs of the model, nor the genetic background.

Aside from these effects, two other QTLs for FHB resistance were detected with a major effect, one on chromosome 2B, and another one on chromosome 7A. The marker *8514068*, in linkage disequilibrium with the QTL on chromosome 7A (Table 5), indicates that the line G8.06 would be the only parental line carrying the resistant allele for this QTL. However, the effect of the QTL was only significant with cross-specific models in the E population, where it resulted in a reduction in FHB severity of 22% on the heads in the field and of 18% on the grains (Table 5). Due to a lack of proximate markers in the chromosomal region of the 7A QTL in the consensus map, the MPP analysis did not detect this QTL. The major effect QTL detected on chromosome 2B was merely polymorphic in the T population where it led to a reduction in field severity of 26% and of 35% on the harvested grains. Epistatic interactions were identified between this QTL and another one of the cross-specific model positioned on the chromosome 6A (Table 5). By refining the analysis using both the bi-allelic and parental models, it was possible to localize this QTL in a reduced area of 1.3 cM on the consensus map, where the 2B QTL effect was further confirmed by cross-validation (Table 7). When aligning the markers of the consensus and T population cross-specific maps of this QTL interval on the wheat physical map, they were located in a 9.6 mega-base pairs (Mbp) region containing 48 high confidence genes. A description of the genes contained in this area and the marker blasting information are summarized in Online Resource 7.

**Fig. 3:**
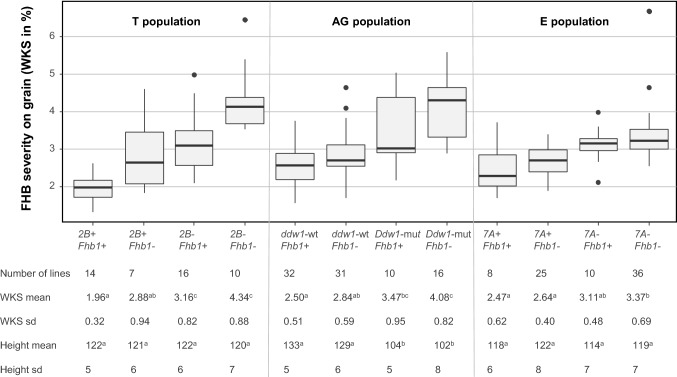
Box plot distributions of F_4_ according to their allele combinations at the two main FHB-resistance loci for each of the three populations based on BLUEs of FHB severity on grain (WKS) calculated across all experiments. Medians are indicated by solid lines, and points represent outliers. For each subgroup, the number of lines, mean values, and standard deviations of FHB severity on grains (WKS) and plant height (cm) are indicated. Values followed by different letters are significantly different (*p* < 0.05) based on the Tukey test performed on each population independently

To illustrate the effects on FHB severity of combining *Fhb1* with other QTLs with major effects on resistance, the lines of the T, AG, and E populations were classified in subgroups according to their allele status at *Fhb1 and* the QTL on 2B for the T population, on 5R for the AG population, and on 7A for the E population. Resistance level and plant height were compared among the different subgroups (Fig. 3, Online resource 8). In the T and AG populations, lines carrying both resistance QTLs had significantly less disease severity than the lines carrying only *Fhb1* and in the AG population, lines carrying the dwarfing allele at *Ddw1* locus were significantly shorter and more susceptible than the ones harboring the wild-type allele.

## Discussion

FHB resistance is a top priority in cereal breeding and is receiving high attention ranging from basic research to cultivar development. Breeding and growing varieties that resist mycotoxin accumulation are of foremost importance for crops such as triticale, which are used primarily on the farm as animal feed, without checking for a potential mycotoxin contamination of the harvest. Additionally, triticale is a useful energy crop for bioethanol fermentation. The typical output of bioethanol production from cereals is that about 1/3 of the cereal mass is converted into bioethanol, 1/3 is converted to CO_2_, and 1/3 is the so-called stillage, which is normally dried to produce DDGS (Distiller’s dried grains with solubles) a coproduct of the distillery industries. DDGS is used as high-value protein feed and could, due to its optimal nutritional composition, partly replace even soygrist in pig fattening (Schedle et al. 2010). Due to the production scheme in bioethanol conversion, mycotoxin contaminations in the starting material are concentrated in the DDGS, by a factor 3 (Schaafsma et al. 2009).

However, relatively little research for FHB resistance has been conducted for triticale until now whereas genetic resistance in bread wheat has been well described. Three related populations between a triticale FHB-resistant donor line with *Fhb1* and *Qfhs.ifa*-*5A* introgressions from bread wheat, and two adapted triticale varieties and one F1 hybrid, were analyzed in this study. Analyzing three mapping populations with large variation in FHB severity allowed further dissecting the genetic basis of FHB resistance in different elite triticale genetic backgrounds and combined QTL detection with QTL validation. Considering the connectivity between these three related populations by using a parental model permitted comparing the effects of QTLs detected in distinct cross-specific models, whereas the use of a bi-allelic model allowed a global characterization of the QTL effects based on all available information and finally improved the quality of their localizations.

### Genetic architecture of FHB resistance in triticale

Even though the disease pressure was significantly different between the four years, no isolate specificity was detected in the genetic architecture of the resistance when comparing the architecture observed in the years 2014–2015 with the architecture observed in the years 2016–2017. The high broad-sense heritability coefficients in the three populations indicate that a large proportion of the variation among line means was due to genetic differences. A total of 9 QTLs with varying effects on FHB resistance were identified on chromosomes 2B, 3B, 5R, 6A, 6B, 7A, and 7B confirming previous results about the quantitative inheritance of FHB resistance in triticale (Dhariwal et al. 2018; Galiano-Carneiro et al. 2019; Kalih et al. 2015; Miedaner et al. 2006a, b; Oettler et al. 2004). Only one QTL was identified on the rye genome Except for the two QTLs on the 6B, all resistant alleles descended from the common parent G8.06, which was preselected for its high resistance to FHB. Significant transgressive segregation was observed in all populations, suggesting the presence of additional resistance QTL which remained undetected, possibly due to the relatively small population sizes. Nevertheless, QTLs with large effects are detectable even in rather small populations (Vales et al. 2005) and four QTLs with the major effect on the resistance to FHB were detected on chromosomes 3B, 2B, 7A, and 5R.

One of the most promising marker–trait associations found in this study was the one identified on chromosome 3B, which mapped in the *Fhb1* region between the SSR markers *gwm493* and *gwm533*. The effects of *Fhb1* observed in our populations were in the same range as the ones previously observed in wheat. Buerstmayr et al. (2003) showed that *Fhb1* explained 20% of phenotypic variance in a spring wheat population, and Prat et al. (2017) reported that it explained 5–14% of the phenotypic variance in three durum wheat populations. In accordance with previous results (Agostinelli et al. 2012; Balut et al. 2013; Buerstmayr et al. 2009; Prat et al. 2017; Pumphrey et al. 2007; Verges et al. 2006), our study showed that the effect of *Fhb1* on improving FHB resistance is robust, but the magnitude may vary depending on the genetic background.

Aside from *Fhb1* two further QTLs on chromosomes 7A and 2B both with the major effect on FHB resistance were detected. Several FHB-resistance QTLs with large effect have been detected in bread wheat on these two chromosomes (Buerstmayr et al. 2009). In 2011, Jayatilake et al. reported a QTL from CS-Sumai 3-7ADSL with a high level of FHB resistance for symptom spread within a spike (type 2) and low deoxynivalenol accumulation in infected kernels (type 3). Designated as *Fhb7AC*, this QTL mapped near the centromere of the chromosome 7A and explained a similar level of resistance than the QTL detected in this study on chromosome 7A (22% phenotypic variation for type 2 and 24% for type 3 resistance, Jayatilake et al. 2011). Further testing will be necessary to uncover whether or not those two QTLs are identical or at proximity. Improvement in the mapping resolution may be a difficult task regarding the proximity with the centromere. The effect of the QTL detected on chromosome 7A in this study, was significant in the E population only, although the closest marker we found in linkage disequilibrium with the QTL (Table 5) indicates that this QTL segregates in all three populations. The cross-specific map built for the E population is 1.5 times larger than the ones of the T and AG populations. This situation did not allow a precise localization of the QTL on the chromosome 7A, and a large physical distance may exist between the QTL and the closest marker. The importance of the QTL effect, associated with the many common markers between the E population map and the one provided by Tyrka et al. (2015), gives us a reasonable level of confidence regarding the presence of this QTL on the chromosome 7A in the E population, but the allele status of the lines Tulus, Agostino, and Grenado is, however, dubious. The parental line El Paso may be the only one carrying the susceptible allele for this QTL, which would explain why the effect of the QTL is significant in the E population only. No report has been found in the literature of any large effect QTL in chromosome 2B coming from populations with Sumai-3 in their pedigree. The parental lines Tulus and Grenado carry the susceptible allele for the QTL on the 2B, which could explain why they were much more susceptible than the other parental lines Agostino, ElPaso, and G8.06. Polymorphism at the QTL locus was detected in the T population only. However, cross-validation results performed with multi-parental models showed comparable level of stability when comparing with *Fhb1*, and both QTLs presented similar additive effects in the T population.

The fourth QTL with the major effect on FHB resistance identified in this study were detected in the AG population, on chromosome 5R, at the exact position where markers linked to the *Ddw1* gene were mapped. It was the only FHB-resistance QTL overlapping with QTL for flowering date and plant height. A large effect of this QTL on plant height and flowering time was previously described in rye and in triticale (Börner et al. 2000; Kalih et al. 2014), while the strong effect of *Ddw1* on FHB resistance was verified in Kalih et al. (2014). It accordingly explained 48%, 77%, and 71% of the genotypic variance for FHB severity, plant height, and flowering time, respectively (Kalih et al. 2014). Similarly, a colocalization for a QTL of FHB resistance and a QTL of plant height was observed on the chromosome 5R by Dhariwal et al. (2018). This QTL was reported to explain 23% of the phenotypic variance for FHB resistance and 13% of the phenotypic variance for plant height (Dhariwal et al. 2018), but the absence of common markers with this study does not allow to draw unambiguous conclusions about its identity with *Ddw1.*

### Association of QTL for FHB resistance and plant height, focusing on *Ddw1*

In this study, we investigated the association of plant height and FHB resistance with specific focus on the dwarfing gene *Ddw1*. The possibility to select for short plant types with high level of FHB resistance is indeed of high interest in cereal breeding. The frequently detected colocalization of QTLs for both traits caused either by linkage or pleiotropy may render the achievement of this breeding goal a difficult task (Buerstmayr et al. 2012; Miedaner and Longin 2014; Prat et al. 2017; Talas et al. 2011). In this study, the level of correlation between plant height and FHB resistance was larger than *r* = 0.5 in the AG population only, which was mainly caused by the effect of *Ddw1*. Given that these two traits were not correlated in the T and E populations, many genotypes matching the breeding goal of high FHB resistance and medium to short stature were found in these two populations (Fig. 1) confirming previous results by Galiano-Carneiro et al. (2019). On the other hand, there was only one short-straw genotype showing high level of resistance associated in the AG population. These results confirm the observations of Kalih et al. (2014) who showed that large population sizes were necessary to identify rare short-straw genotypes due to the dwarfing allele of *Ddw1* with an acceptably high level of FHB resistance.

### Introgressing wheat resistance factors in elite triticale, a promising path for enhancing FHB resistance of triticale

Crossing hexaploid triticale with hexaploid wheat, and backcrossing to triticale, has been extensively used in the triticale breeding history and tends to produce natural hexaploid triticale with frequent translocations observed from the D genome toward the R genome (Jenkins 1969; Kiss 1966; Lukaszewski and Gustafson 1983; Merker 1975; Sanchez-Monge 1958). With 7 resistance alleles on the 9 QTLs detected, including those of the 4 major effect QTLs, the line G8.06 harbors a very promising QTL combination. The digital phenotyping methods used in this study enabled a characterization of type 4 resistance. Whether the FHB resistance observed in the field was due mainly to type 1 or type 2 resistance warrants further investigations. Although both major wheat resistance factors from the ancestral bread wheat line CM-82036, *Qfhs.ifa*-*5A*, and *Fhb1,* (Buerstmayr et al. 2003) were polymorphic in the three tested populations, no significant effect was found for *Qfhs.ifa*-*5A.* Steiner et al. (2019) discovered that *Qfhs.ifa*-*5A* improves resistance to initial infection most likely through a passive resistance mechanism by enhancing anther extrusion in wheat. The very high extent of anther extrusion typical for triticale may therefore mask the effect of this QTL. By contrast, the use of three related populations has allowed for the first time the detection and the validation of *Fhb1* in triticale. The recent genome-wide association study performed on a panel of 133 diverse winter triticale cultivars and elite breeding lines by Galiano-Carneiro et al. (2019) did not disclose any FHB resistance QTL on the chromosome 3B. This possibly shows that *Fhb1* was absent in the triticale genepool and that the novel germplasm developed for our study is the first triticale breeding material with *Fhb1* introgressed.

### Whitened kernel surface (WKS), a novel digital trait for scoring FHB resistance

Due to the complexity of resistance phenomena, the genetic architecture of resistance may vary depending of the specificities of the phenotyping method used for its evaluation. In this study, FHB resistance was evaluated for two FHB related traits. The first one assessed FHB symptom severity on a whole plot basis in the field (AUDPC) which encompasses an integrated measure for FHB severity but does not distinguish types of resistance in the sense of Schroeder and Christensen (1963). The second one was based on the severity of symptoms on grains measured by WKS which is a measure for resistance to kernel infection, also called type 4 in the sense of Mesterházy (1995). Notably, similar genetic architecture of the resistance was observed for both traits, AUDPC and WKS, in the three tested populations. The four QTLs with the major effect on resistance to FHB were detectable with both the traditional field severity evaluation (AUDPC) as well as WKS. Similar LOD values were observed for *Fhb1*, *Ddw1*, and the QTL on the chromosome 7A for both traits. Higher heritability coefficients were found for WKS compared to AUDPC. WKS scoring allows measuring symptoms on many samples in an easier way than field scoring, and Ollier et al. (2018) showed that WKS displays high correlations with the mycotoxin content.

### Perspective for triticale breeding and conclusions

One of the main outcomes of this project was the detection and the validation for the first time, of *Fhb1* in a triticale background, which represents a significant step forward in improving FHB resistance for this crop. Surprisingly, despite a high effect on resistance, *Fhb1* has not yet been deployed in commercial small-grain cereal cultivars by European breeders (Steiner et al. 2017). The agronomic features of Sumai-3 and CM-82036 that are very far from high-yielding elite breeding germplasms may be one of the main issues which hampered this introgression. The two steps of backcrossing with Santop, and the successive crosses with triticale elite cultivars that were realized in this study, enabled the development of novel FHB-resistant genotypes that are agronomically closer to modern European germplasm. These genotypes represent improved germplasm for continuing a pre-breeding process targeting an introgression of *Fhb1* in elite winter triticale cultivars. As an example, nine triticale lines with beneficial QTL combinations for FHB resistance and very high level of resistance for both traits, AUDPC and WKS, have been identified, and appear attractive for future research and pre-breeding purposes (Online resource 9). They represent excellent candidates for enhancing FHB resistance in practical triticale breeding programs, and with seven resistant alleles on nine QTLs detected, the breeding line and common parent of our population, G8.06, represents by itself a valuable genetic resource for triticale breeding.

Aside from *Fhb1* three further QTLs on chromosomes 7A, 2B, and 5R all with the major effect on FHB resistance were detected. The difficulty to identify markers in segregation with the QTL detected on chromosome 7A possibly restrains the use of this QTL in a breeding program despite its high effect on resistance. On the contrary, the QTL on chromosome 2B appears particularly interesting for marker-assisted breeding and gene cloning. It was mapped with a much greater precision than the QTL on chromosome 7A and localized in a marker rich area, which enable the identification of diagnostic markers associated with the QTL. However, this original resistance factor with the major effect on the FHB resistance still needs to be validated in different breeding material.

Regarding the use of the dwarfing gene *Ddw1* on the chromosome 5R in triticale breeding programs, wheat breeders used to select first for lines with dwarfing alleles, in particular *Rht* genes and then compensate their negative effect on FHB resistance by pyramiding other resistance QTL (Lu et al. 2011; Prat et al. 2017). This strategy is appropriate, knowing that *Rht* genes have a positive impact on yield, whereas Alheit et al. (2011) concluded that the dwarfing gene *Ddw1* reduced grain yield in triticale. Hence, it may be more advantageous for triticale breeders to conserve the tall allele of *Ddw1* in their breeding lines and reduce the impact on stature by using other plant height QTLs which do not have an impact on the resistance as for example the QTL we have identified on the chromosome 5A.

Those four QTLs with the major effect on the resistance to FHB constitute promising candidates for improving resistance in triticale. The strong population effect characterized for *Fhb1* is a frequent feature for FHB-resistant QTLs (Pumphrey et al. 2007) and may be explained by numerous additional QTLs with minor effects and interactions with the genetic background. By taking into account the entire genome with both QTL with minor and major effects on resistance, genomic selection may be a useful strategy for FHB-resistance breeding, rather than simple marker-assisted selection (MAS) based on few QTL with the major effect only. Some preliminary results are available and appear promising (Arruda et al. 2015, 2016; Galiano-Carneiro et al. 2019; Steiner et al. 2017; Würschum et al. 2017), but other publications have concluded that genomic selection only slightly improved predictive ability compared to marker-assisted selection (Miedaner et al. 2017) or even led to lower accuracies than using QTL targeted markers alone (Rutkoski et al. 2012). Even so, marker-assisted selection has already demonstrated its efficiency for improving FHB resistance in wheat (Anderson et al. 2007; Miedaner et al. 2006a, b; Salameh et al. 2011; Wilde et al. 2007) and could therefore be a more affordable option for triticale breeding programs in which high-density fingerprinting is not commonly implemented. Finally, the new scoring method based on digital evaluation of the whitened kernel surface (WKS) appears as an efficient and flexible tool to enable FHB-resistance scoring and a large-scale identification of breeding lines with low risk of mycotoxin contamination.

## Electronic supplementary material

Below is the link to the electronic supplementary material.

Supplementary material 1 (PDF 112 kb)

Supplementary material 2 (PDF 205 kb)

Supplementary material 3 (PDF 97 kb)

Supplementary material 4 (PDF 156 kb)

Supplementary material 5 (XLSX 87 kb)

Supplementary material 6 (PDF 122 kb)

Supplementary material 7 (XLSX 21 kb)

Supplementary material 8 (PPTX 58 kb)

Supplementary material 9 (XLSX 15 kb)
