# Supplementary material for: QTL mapping and successful introgression of the spring wheat-derived QTL Fhb1 for Fusarium head blight resistance in three European triticale populations
Source: Theor Appl Genet. 2020 Jan 20;133(2):457–77. doi: 10.1007/s00122-019-03476-0 (PMC6985197; doi:10.1007/s00122-019-03476-0)

Article title: QTL mapping and successful introgression of the spring wheat derived QTL *Fhb1* for Fusarium head blight resistance in three European triticale populations

Journal: Theoritical Applied Genetics

Authors: Ollier Marine[1236], Talle Vincent[1], Brisset Anne-Laure[1], Le Bihan Zoé[1], Duerr Simon[15], Lemmens Marc[1], Goudemand Ellen[3], Robert Olivier[34], Hilbert Jean-Louis[2], Buerstmayr Hermann[1]

1. BOKU-University of Natural Resources and Life Sciences Vienna, Department of Agrobiotechnology, IFA-Tulln, Institute of Biotechnology in Plant Production, Konrad Lorenz Str. 20, 3430 Tulln, Austria
2. EA 7394, USC INRA 1411, Institut Charles Viollette (ICV), Agro-food and biotechnology research institute, Université de Lille, INRA, ISA, Univ. Artois, Univ. Littoral Côte d'Opale, Cité Scientifique, 59655 Villeneuve d'Ascq, France
3. Florimond-Desprez Veuve & Fils SAS, 3 rue Florimond-Desprez, BP 41, 59242 Cappelle-en-Pévèle, France
4. Deceased on February 21, 2017
5. Current address: Saatzucht Donau GmbH & Co KG, Breeding Station Reichersberg, Austria.
6. Current address: Bayer Crop Science, Le petit Boissay, Toury, France.

Corresponding author: Marine Ollier, marine.ollier@bayer.com

ESM_1 : Overview of the climatic conditions observed at IFA-Tulln (Austria) for the years 2014 to 2017, with the level of precipitation (mm) draw in solid line, and the average temperature (°C) draw in dashed line.

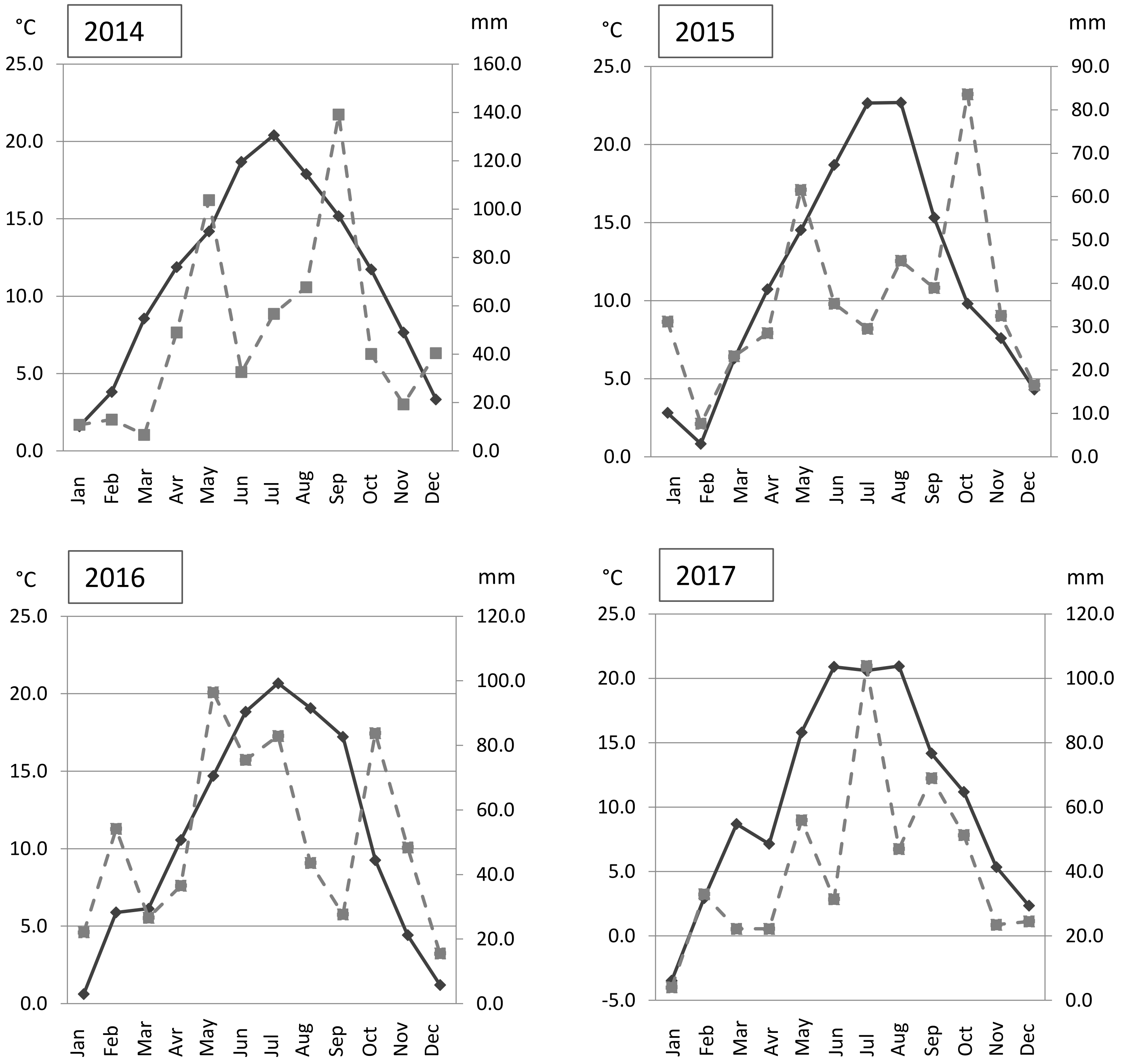

Supplement: Supplementary file 1 — Supplementary material 1 (PDF 112 kb) [file 122_2019_3476_MOESM1_ESM.pdf]
