# Supplementary material for: QTL mapping and successful introgression of the spring wheat-derived QTL Fhb1 for Fusarium head blight resistance in three European triticale populations
Source: Theor Appl Genet. 2020 Jan 20;133(2):457–77. doi: 10.1007/s00122-019-03476-0 (PMC6985197; doi:10.1007/s00122-019-03476-0)
Supplement: Supplementary file 3 — Supplementary material 3 (PDF 97 kb) [file 122_2019_3476_MOESM3_ESM.pdf]

Article title: QTL mapping and successful introgression of the spring wheat derived QTL *Fhb1* for Fusarium head blight resistance in three European triticale populations

Journal: Theoritical Applied Genetics

Authors: Ollier Marine[1236], Talle Vincent[1], Brisset Anne-Laure[1], Le Bihan Zoé[1], Duerr Simon[15], Lemmens Marc[1], Goudemand Ellen[3], Robert Olivier[34], Hilbert Jean-Louis[2], Buerstmayr Hermann[1]

1. BOKU-University of Natural Resources and Life Sciences Vienna, Department of Agrobiotechnology, IFA-Tulln, Institute of Biotechnology in Plant Production, Konrad Lorenz Str. 20, 3430 Tulln, Austria
2. EA 7394, USC INRA 1411, Institut Charles Viollette (ICV), Agro-food and biotechnology research institute, Université de Lille, INRA, ISA, Univ. Artois, Univ. Littoral Côte d'Opale, Cité Scientifique, 59655 Villeneuve d'Ascq, France
3. Florimond-Desprez Veuve & Fils SAS, 3 rue Florimond-Desprez, BP 41, 59242 Cappelle-en-Pévèle, France
4. Deceased on February 21, 2017
5. Current address: Saatzucht Donau GmbH & Co KG, Breeding Station Reichersberg, Austria.
6. Current address: Bayer Crop Science, Le petit Boissay, Toury, France.

Corresponding author: Marine Ollier, marine.ollier@bayer.com

ESM3 Variance component estimates of genotype $\sigma^2_{Genotype}$, year σ^2_{Year}, block within year $\sigma^2_{Block\ within\ Year}$, genotype × year $\sigma^2_{Genotype\ x\ Year}$ and the residual effects σ^2 error for FHB severity (AUDPC, WKS), plant height and flowering date across three experiments for populations T, AG, E

Tulus x G8.06 (T) population

Trait	Variance component				
	$\sigma^2_{Genotype}$	σ^2_{Year}	$\sigma^2_{Block\ within\ Year}$	$\sigma^2_{Genotype\times Year}$	σ^2_{error}
FHB severity (AUDPC)	4.41E+03	1.13E+05	9.17E+02	2.26E+03	8.39E+03
FHB severity (WKS)	9.79E-01	3.53E-07	1.40E-01	2.68E-01	3.68E-01
Plant height	3.17E+01	9.47E+01	1.09E+01	2.06E+00	2.87E+01
Flowering date	8.99E-01	6.54E+00	4.14E+00	4.10E-16	1.20E+00

(Agostino x Grenado) x G8.06 (AG) population

Trait	Variance component				
	$\sigma^2_{Genotype}$	σ^2_{Year}	$\sigma^2_{Block\ within\ Year}$	$\sigma^2_{Genotype\times Year}$	σ^2_{error}
FHB severity (AUDPC)	5.77E+03	1.29E+05	3.11E+03	3.35E+03	7.02E+03
FHB severity (WKS)	6.93E-01	<1.0E-8	3.13E-01	1.08E-01	3.21E-01
Plant height	2.19E+02	9.62E+01	2.05E+01	1.68E+01	2.04E+01
Flowering date	5.76E-01	9.57E+00	1.69E+00	1.28E-01	9.87E-01

El Paso x G8.06 (E) population

Trait	Variance component				
	$\sigma^2_{Genotype}$	σ^2_{Year}	$\sigma^2_{Block\ within\ Year}$	$\sigma^2_{Genotype\times Year}$	σ^2_{error}
FHB severity (AUDPC)	2.89E+03	4.47E+04	3.86E+03	1.41E+03	5.33E+03
FHB severity (WKS)	3.62E-01	8.92E-02	1.37E-01	7.21E-02	2.19E-01
Plant height	5.16E+01	8.34E+01	2.14E+01	1.25E+00	2.66E+01
Flowering date	6.26E-01	7.84E+00	1.97E+00	2.00E-10	1.11E+00
