# Supplementary material for: QTL mapping and successful introgression of the spring wheat-derived QTL Fhb1 for Fusarium head blight resistance in three European triticale populations
Source: Theor Appl Genet. 2020 Jan 20;133(2):457–77. doi: 10.1007/s00122-019-03476-0 (PMC6985197; doi:10.1007/s00122-019-03476-0)

Article title : QTL mapping and successful introgression of the spring wheat derived QTL *Fhb1* for Fusarium head blight resistance in three European triticale populations

Journal: Theoritical Applied Genetics

Authors: Ollier Marine[1236], Talle Vincent[1], Brisset Anne-Laure[1], Le Bihan Zoé[1], Duerr Simon[15], Lemmens Marc[1], Goudemand Ellen[3], Robert Olivier[34], Hilbert Jean-Louis[2], Buerstmayr Hermann[1]

1. BOKU-University of Natural Resources and Life Sciences Vienna, Department of Agrobiotechnology, IFA-Tulln, Institute of Biotechnology in Plant Production, Konrad Lorenz Str. 20, 3430 Tulln, Austria
2. EA 7394, USC INRA 1411, Institut Charles Viollette (ICV), Agro-food and biotechnology research institute, Université de Lille, INRA, ISA, Univ. Artois, Univ. Littoral Côte d'Opale, Cité Scientifique, 59655 Villeneuve d'Ascq, France
3. Florimond-Desprez Veuve & Fils SAS, 3 rue Florimond-Desprez, BP 41, 59242 Cappelle-en-Pévèle, France
4. Deceased on February 21, 2017
5. Current address: Saatzucht Donau GmbH & Co KG, Breeding Station Reichersberg, Austria.
6. Current address: Bayer Crop Science, Le petit Boissay, Toury, France.

Corresponding author: Marine Ollier, marine.ollier@bayer.com

ESM_4 : Linkage maps and positions of the four QTL with major effect on FHB severity and coinciding morphological traits in the three populations based on BLUEs calculated across all experiments. For readability, only selected markers are shown. Loci closest to the QTL peak of FHB severity are in bold. QTL bars span an LOD drop of 1.5 from maximum LOD.

Page 1/2

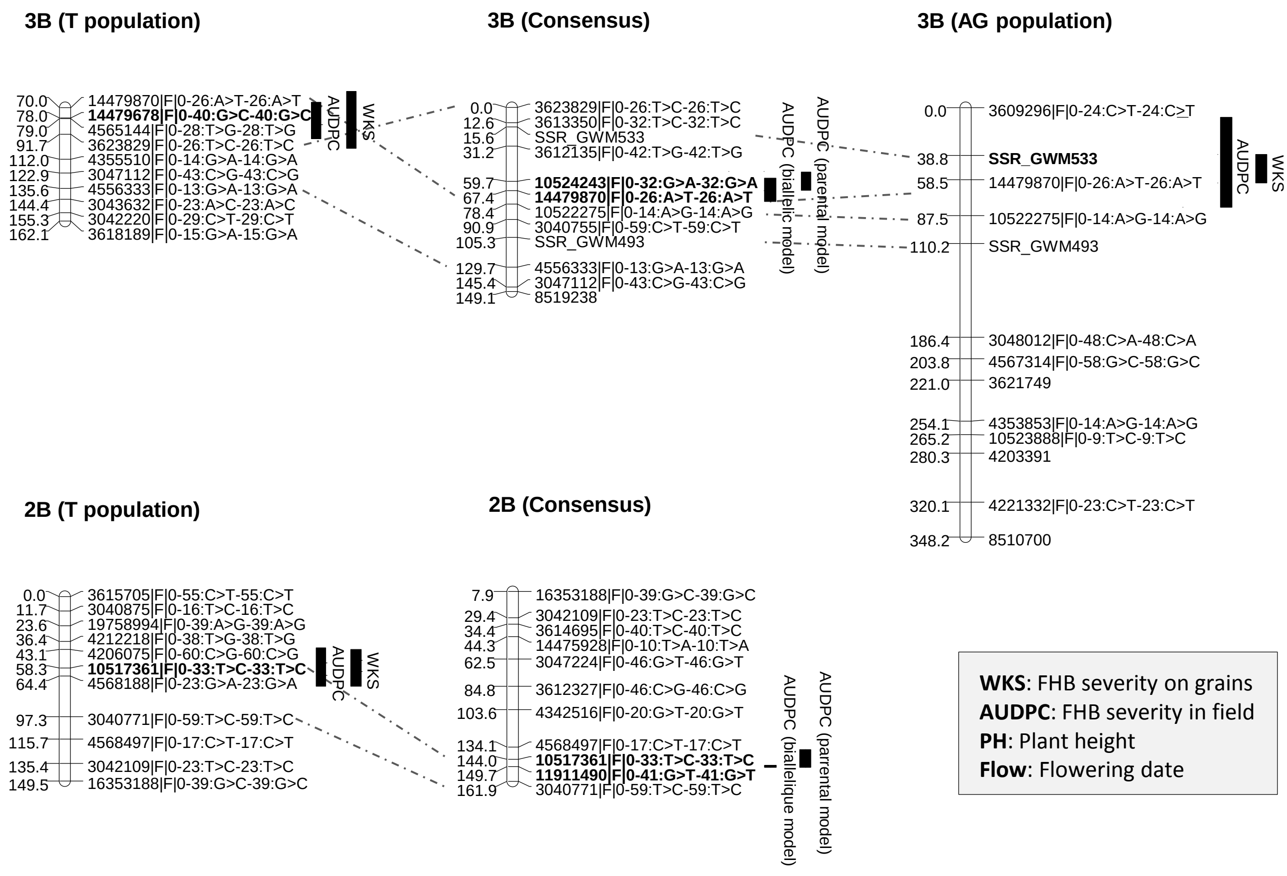

ESM_4 : Linkage maps and positions of the four QTL with major effect on FHB severity and coinciding morphological traits in the three populations based on BLUEs calculated across all experiments. For readability, only selected markers are shown. Loci closest to the QTL peak of FHB severity are in bold. QTL bars span an LOD drop of 1.5 from maximum LOD.

Page 2/2

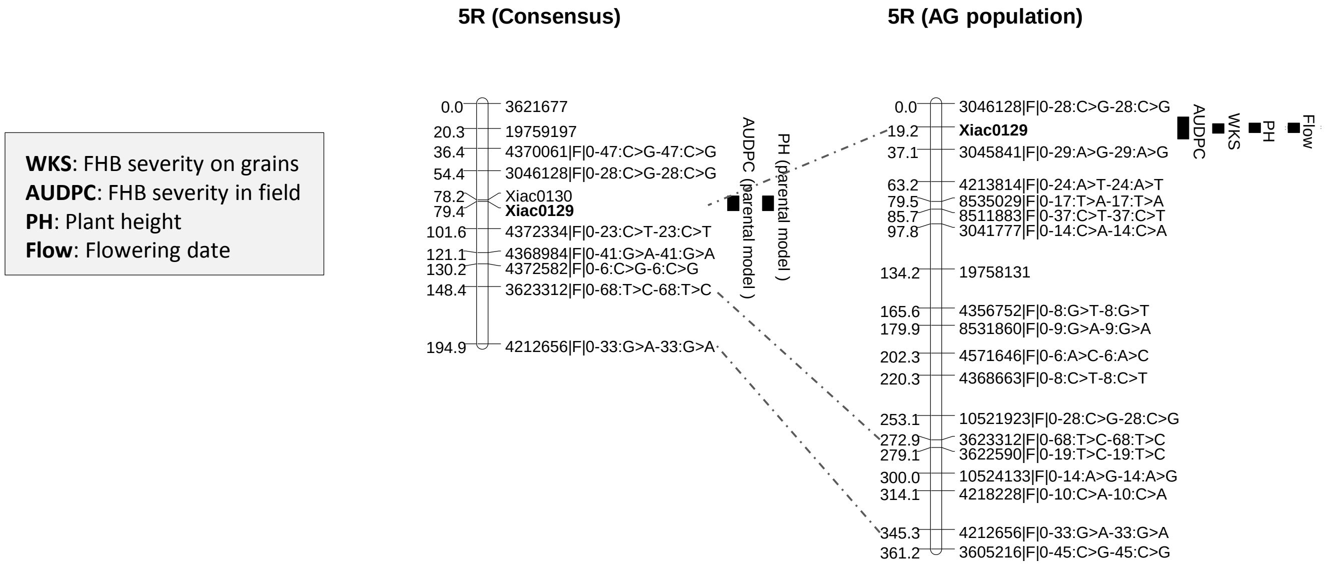

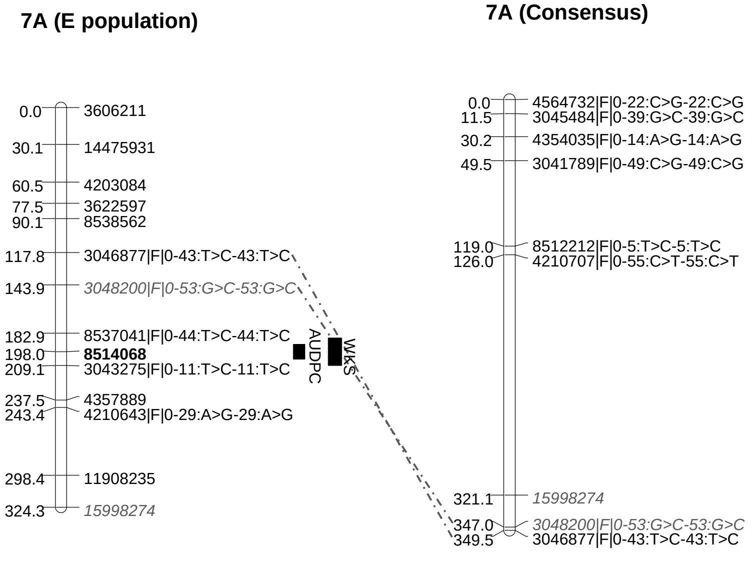

Supplement: Supplementary file 4 — Supplementary material 4 (PDF 156 kb) [file 122_2019_3476_MOESM4_ESM.pdf]
