# Supplementary material for: QTL mapping and successful introgression of the spring wheat-derived QTL Fhb1 for Fusarium head blight resistance in three European triticale populations
Source: Theor Appl Genet. 2020 Jan 20;133(2):457–77. doi: 10.1007/s00122-019-03476-0 (PMC6985197; doi:10.1007/s00122-019-03476-0)
Supplement: Supplementary file 6 — Supplementary material 6 (PDF 122 kb) [file 122_2019_3476_MOESM6_ESM.pdf]

Article title: QTL mapping and successful introgression of the spring wheat derived QTL *Fhb1* for Fusarium head blight resistance in three European triticale populations

Journal: Theoritical Applied Genetics

Authors: Ollier Marine[1236], Talle Vincent[1], Brisset Anne-Laure[1], Le Bihan Zoé[1], Duerr Simon[15], Lemmens Marc[1], Goudemand Ellen[3], Robert Olivier[34], Hilbert Jean-Louis[2], Buerstmayr Hermann[1]

1. BOKU-University of Natural Resources and Life Sciences Vienna, Department of Agrobiotechnology, IFA-Tulln, Institute of Biotechnology in Plant Production, Konrad Lorenz Str. 20, 3430 Tulln, Austria
2. EA 7394, USC INRA 1411, Institut Charles Viollette (ICV), Agro-food and biotechnology research institute, Université de Lille, INRA, ISA, Univ. Artois, Univ. Littoral Côte d'Opale, Cité Scientifique, 59655 Villeneuve d'Ascq, France
3. Florimond-Desprez Veuve & Fils SAS, 3 rue Florimond-Desprez, BP 41, 59242 Cappelle-en-Pévèle, France
4. Deceased on February 21, 2017
5. Current address: Saatzucht Donau GmbH & Co KG, Breeding Station Reichersberg, Austria.
6. Current address: Bayer Crop Science, Le petit Boissay, Toury, France.

Corresponding author: Marine Ollier, marine.ollier@bayer.com

ESM_6 : Box plot distributions of F_4 according to their alleles at *Fhb1* and *Qfhs.ifa-5A* loci for the three tested populations based on BLUEs of FHB severity in field (AUDPC). BLUEs were calculated across all experiments. Medians are indicated by solid lines, points represent outliers. For each subgroup, the number of lines, mean values and standard deviations FHB severity in field (AUDPC) are indicated. Values followed by different letters are significantly different ($p < 0.05$) based on Tukey test performed on each population independently.

Page 1/2

ESM_6 : Box plot distributions of F_4 according to their alleles at *Fhb1* and *Qfhs.ifa-5A* loci for the three tested populations based on BLUEs of FHB severity in field (AUDPC). BLUEs were calculated across all experiments. Medians are indicated by solid lines, points represent outliers. For each subgroup, the number of lines, mean values and standard deviations FHB severity in field (AUDPC) are indicated. Values followed by different letters are significantly different ($p < 0.05$) based on Tukey test performed on each population independently.

Page 2/2

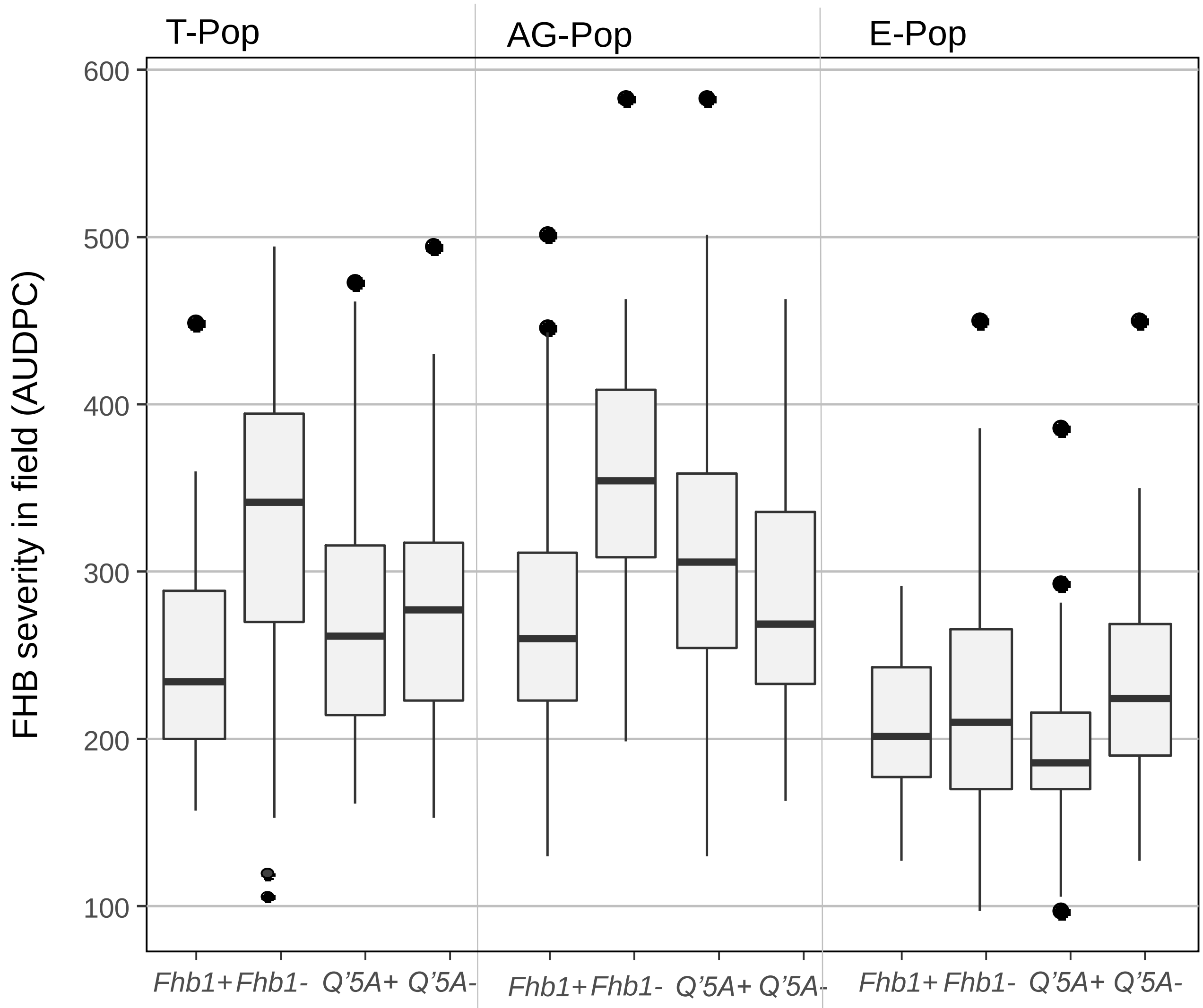

Number of lines73	39	23	39	39	42	30	46	42	23	54	42	34
AUDPC mean	251[a]	335[b]	277[ab]	280[ab]	279[a]	356[b]	312[ab]	285[ab]	208[a]	222[a]	198[a]	235[a]
AUDPC sd	63	88	81	77	77	82	88	69	43	73	57	65
