# Supplementary material for: QTL mapping and successful introgression of the spring wheat-derived QTL Fhb1 for Fusarium head blight resistance in three European triticale populations
Source: Theor Appl Genet. 2020 Jan 20;133(2):457–77. doi: 10.1007/s00122-019-03476-0 (PMC6985197; doi:10.1007/s00122-019-03476-0)
Supplement: Supplementary file 8 — Supplementary material 8 (PPTX 58 kb) [file 122_2019_3476_MOESM8_ESM.pptx]

## Slide 1
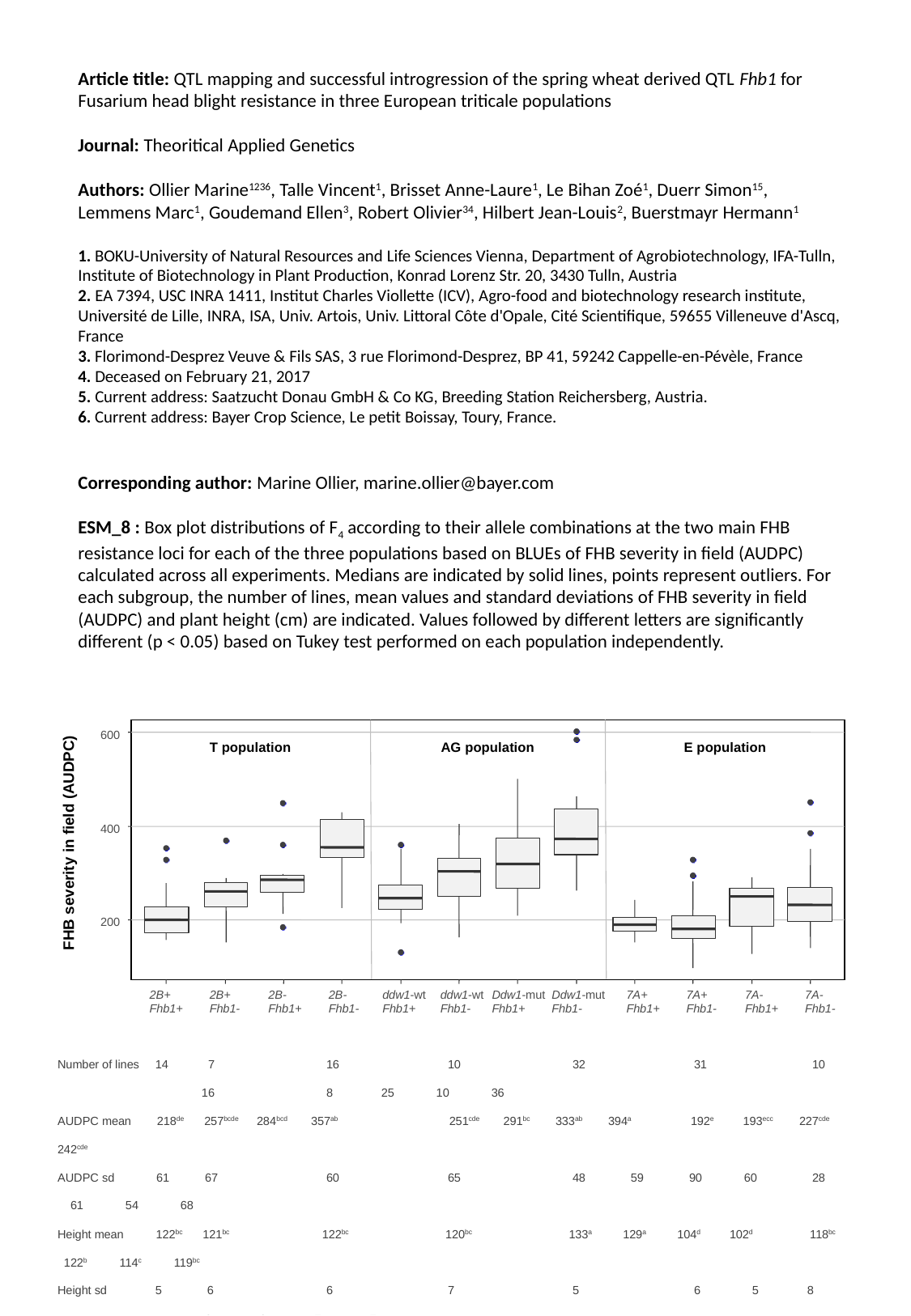

Article title: QTL mapping and successful introgression of the spring wheat derived QTL Fhb1 for Fusarium head blight resistance in three European triticale populations
Journal: Theoritical Applied Genetics
Authors: Ollier Marine1236, Talle Vincent1, Brisset Anne-Laure1, Le Bihan Zoé1, Duerr Simon15, Lemmens Marc1, Goudemand Ellen3, Robert Olivier34, Hilbert Jean-Louis2, Buerstmayr Hermann1
1. BOKU-University of Natural Resources and Life Sciences Vienna, Department of Agrobiotechnology, IFA-Tulln, Institute of Biotechnology in Plant Production, Konrad Lorenz Str. 20, 3430 Tulln, Austria
2. EA 7394, USC INRA 1411, Institut Charles Viollette (ICV), Agro-food and biotechnology research institute, Université de Lille, INRA, ISA, Univ. Artois, Univ. Littoral Côte d'Opale, Cité Scientifique, 59655 Villeneuve d'Ascq, France
3. Florimond-Desprez Veuve & Fils SAS, 3 rue Florimond-Desprez, BP 41, 59242 Cappelle-en-Pévèle, France
4. Deceased on February 21, 2017
5. Current address: Saatzucht Donau GmbH & Co KG, Breeding Station Reichersberg, Austria.
6. Current address: Bayer Crop Science, Le petit Boissay, Toury, France.
Corresponding author: Marine Ollier, marine.ollier@bayer.com
ESM_8 : Box plot distributions of F4 according to their allele combinations at the two main FHB resistance loci for each of the three populations based on BLUEs of FHB severity in field (AUDPC) calculated across all experiments. Medians are indicated by solid lines, points represent outliers. For each subgroup, the number of lines, mean values and standard deviations of FHB severity in field (AUDPC) and plant height (cm) are indicated. Values followed by different letters are significantly different (p < 0.05) based on Tukey test performed on each population independently.
600
T population
E population
AG population
400
FHB severity in field (AUDPC)
200
2B+
Fhb1+
2B+
Fhb1-
2B-
Fhb1+
2B-
Fhb1-
ddw1-wt
Fhb1+
ddw1-wt
Fhb1-
Ddw1-mut
Fhb1+
Ddw1-mut
Fhb1-
7A+
Fhb1+
7A+
Fhb1-
7A-
Fhb1+
7A-
Fhb1-
Number of lines 14	 7	 16	 10	 32	 31	 10	 16	 8 25 10 36
AUDPC mean 218de 257bcde 284bcd 357ab	 251cde 291bc 333ab 394a 	 192e 193ecc 227cde 242cde
AUDPC sd 61 67	 60	 65 	 48 59 90 60 	 28 61 54 68
Height mean 122bc 121bc	 122bc	 120bc 	 133a 129a 104d 102d	 118bc 122b 114c 119bc
Height sd 5 6	 6	 7 	 5 	 6 5 8	 6 8 7 7
